# Organic metabolite uptake by diazotrophs in the North Pacific Ocean

**DOI:** 10.1093/ismeco/ycaf061

**Published:** 2025-05-05

**Authors:** Alba Filella, Aurélie Cébron, Benoît Paix, Marine Vallet, Pauline Martinot, Léa Guyomarch, Catherine Guigue, Marc Tedetti, Olivier Grosso, Kendra A Turk-Kubo, Lasse Riemann, Mar Benavides

**Affiliations:** Aix-Marseille Université, Université de Toulon, Centre National de la Recherche Scientifique (CNRS), Institut de Recherche pour le Développement (IRD), Mediterranean Institute of Oceanology (MIO) UM 110, 13288, Marseille, France; Turing Centre for Living Systems, Aix-Marseille University, 13009, Marseille, France; Department of Molecular and Cellular Biology, The University of Arizona, Tucson, AZ 85721, United States; Université de Lorraine, CNRS, Laboratoire Interdisciplinaire des Environnements Continentaux (LIEC), 70239, Nancy, France; Marine Biodiversity group, Alpine Center for research on trophic networks and limnic ecosystems (UMR CARRTEL), Institut National de Recherche en Agriculture, Alimentation et Environnement (INRAE) -Université Savoie Mont-Blanc, 74200, Thonon-les-Bains, France; Max Planck Fellow Group Plankton Community Interactions, Max Planck Institute for Chemical Ecology, 07745, Jena, Germany; Institute for Inorganic and Analytical Chemistry, Friedrich Schiller University of Jena, 07743, Jena, Germany; Aix-Marseille Université, Université de Toulon, Centre National de la Recherche Scientifique (CNRS), Institut de Recherche pour le Développement (IRD), Mediterranean Institute of Oceanology (MIO) UM 110, 13288, Marseille, France; Aix-Marseille Université, Université de Toulon, Centre National de la Recherche Scientifique (CNRS), Institut de Recherche pour le Développement (IRD), Mediterranean Institute of Oceanology (MIO) UM 110, 13288, Marseille, France; Aix-Marseille Université, Université de Toulon, Centre National de la Recherche Scientifique (CNRS), Institut de Recherche pour le Développement (IRD), Mediterranean Institute of Oceanology (MIO) UM 110, 13288, Marseille, France; Aix-Marseille Université, Université de Toulon, Centre National de la Recherche Scientifique (CNRS), Institut de Recherche pour le Développement (IRD), Mediterranean Institute of Oceanology (MIO) UM 110, 13288, Marseille, France; Aix-Marseille Université, Université de Toulon, Centre National de la Recherche Scientifique (CNRS), Institut de Recherche pour le Développement (IRD), Mediterranean Institute of Oceanology (MIO) UM 110, 13288, Marseille, France; Ocean Sciences Department, University of California, Santa Cruz, CA 95064, United States; Department of Biology, University of Copenhagen, 2200, Copenhagen, Denmark; Aix-Marseille Université, Université de Toulon, Centre National de la Recherche Scientifique (CNRS), Institut de Recherche pour le Développement (IRD), Mediterranean Institute of Oceanology (MIO) UM 110, 13288, Marseille, France; Turing Centre for Living Systems, Aix-Marseille University, 13009, Marseille, France; Ocean BioGeosciences Group, National Oceanography Centre (NOC), SO14 3ZH, Southampton, United Kingdom

**Keywords:** DNA-SIP, *nifH*, osmotrophy, N_2_ fixation, DOM, DOM composition, mixotrophy, NCDs

## Abstract

Dinitrogen (N₂) fixation by diazotrophs supports ocean productivity. Diazotrophs include photoautotrophic cyanobacteria, non-cyanobacterial diazotrophs (NCDs), and the recently discovered N_2_-fixing haptophyte. While NCDs are ubiquitous in the ocean, their ecology and metabolism remain largely unknown. Unlike cyanobacterial diazotrophs and the haptophyte, NCDs are primarily heterotrophic and depend on dissolved organic matter (DOM) for carbon and energy. However, conventional DOM amendment incubations do not allow discerning how different diazotrophs use DOM molecules, limiting our knowledge on DOM–diazotroph interactions. To identify diazotrophs using DOM, we amended North Pacific microbial communities with ^13^C-labeled DOM from phytoplankton cultures that was molecularly characterized, revealing the dominance of nitrogen-rich compounds. After DOM additions, we observed a community shift from cyanobacterial diazotrophs like *Crocosphaera* and *Trichodesmium* to NCDs at stations where the N_2_-fixing haptophyte abundance was relatively low. Through DNA stable isotope probing and gene sequencing, we identified diverse diazotrophs capable of taking up DOM. Our findings highlight unexpected DOM uptake by the haptophyte’s nitroplast, changes in community structure, and previously unrecognized osmotrophic behavior in NCDs, shaped by local biogeochemical conditions.

## Introduction

Marine microorganisms called diazotrophs fix dinitrogen (N_2_) into ammonium, providing a critical source of reactive nitrogen in marine ecosystems. Research has traditionally focused on cyanobacterial diazotroph species such as the filamentous *Trichodesmium*, the unicellular *Crocosphaera*, and UCYN-A (e.g. [1–3]), recently reconsidered as an early-stage organelle (the “nitroplast”) of the haptophyte *Braarudosphaera bigelowii* [4]. However, non-cyanobacterial diazotrophs (NCDs) have a broader distribution than cyanobacterial diazotrophs in marine ecosystems, often representing the largest proportion of the community based on nitrogenase gene (*nifH*) amplicon sequencing [5, 6]. Still, the contribution of NCDs to N_2_ fixation inputs remains poorly constrained [7].

Contrary to cyanobacterial diazotrophs and the N_2_-fixing *B. bigelowii* that obtain carbon and energy from photosynthesis, metagenome-assembled genomes (MAGs) indicate that NCDs have the genetic machinery to obtain carbon, nutrients, and energy from organic matter through a wide range of metabolic strategies, including photo- and chemoheterotrophy [8–11]. Several studies have reported enhanced bulk N_2_ fixation rates, *nifH* gene expression, and growth of NCDs in response to dissolved organic matter (DOM) additions, including proteobacteria and Cluster-III taxa [12–14]. However, cyanobacterial diazotrophs also respond to DOM additions with enhanced growth rates and *nifH* gene expression (e.g. [13, 15–19]), suggesting that DOM affects N_2_ fixation inputs by both cyanobacterial and NCDs.

By controlling nitrogen availability in vast ocean regions, diazotrophs sustain marine productivity and contribute to carbon sequestration and the regulation of climate [20]. In turn, climate change-induced stresses on diazotrophs, such as decreased activity under high temperatures and low pH, can be alleviated by DOM uptake [21]. Investigating DOM–diazotroph interactions is needed to improve our understanding of their current and future role as key nitrogen suppliers. This can be a daunting task due to the high molecular complexity of DOM [22]. Our current understanding of DOM–diazotroph interactions is based on incubation experiments where field or cultured diazotrophs are incubated with relatively simple DOM molecules such as glucose or mannitol, which do not reflect the complexity of the marine DOM pool [22–24]. Marine DOM is mainly produced by phytoplankton photosynthates, subsequently consumed and transformed by heterotrophs and altered by abiotic factors such as solar radiation [25–27]. As a result, labile DOM only represents 0.03% of the total dissolved organic carbon contained in marine DOM (662.2 Pg C; 28). The chemical composition of DOM is not fully known. However, techniques such as ultra-high resolution mass spectrometry have identified >20 000 molecular formulas with >30 isomers each, totaling >600 000 compounds, although marine DOM may contain several million distinct organic compounds [28–30].

Given the wide diversity of both diazotroph species and DOM compounds, establishing links between them has proven challenging (e.g. 14, 16). Indirect approaches such as measuring bulk N_2_ fixation rates in response to DOM additions integrate the signals from the entire diazotroph community and cannot resolve which diazotroph taxa are actively consuming DOM compounds. Deoxyribonucleic acid (DNA) Stable-Isotope Probing (DNA-SIP) offers a means of tracing isotopically labeled substrates into DNA, allowing microbial identity to be linked to catabolic activity [31]. Here, we investigate the uptake of phytoplankton-derived DOM by diazotroph communities in the North Pacific Ocean. Using DNA-SIP with a molecularly characterized DOM substrate, we provide direct evidence of DOM uptake by different diazotrophic taxa. Our results suggest that DOM plays an essential role for photoautotrophic and chemoorganoheterotrophic diazotrophs alike, revealing novel osmotrophic metabolisms and ecological strategies allowing them to thrive under unfavorable conditions and expand their traditional niche.

## Materials and methods

### Experimental design and sampling procedure

**Figure 1:**
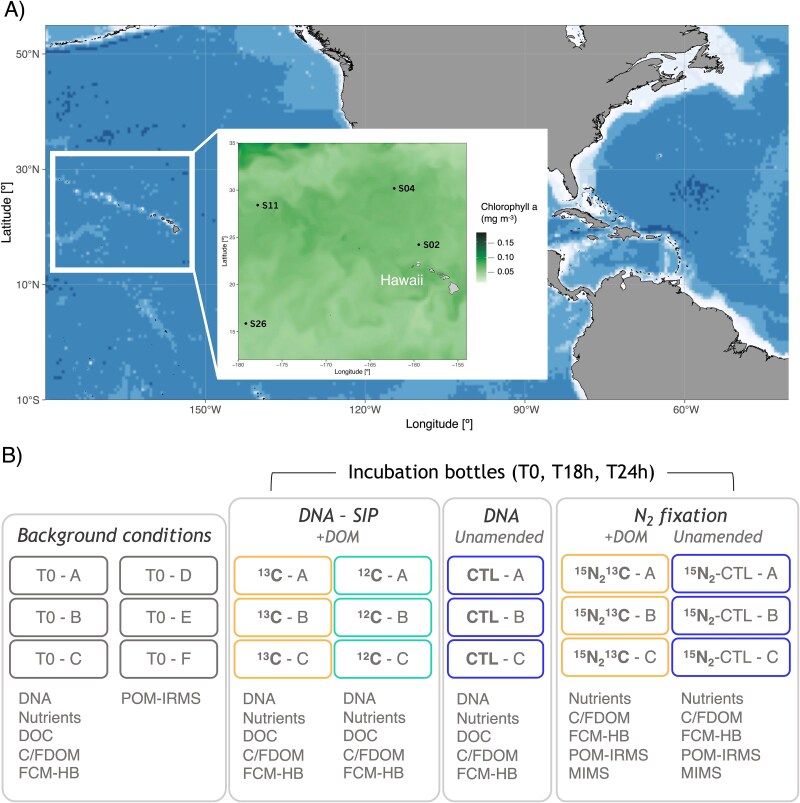
Surface (10 m) chlorophyll-*a* station map (A) and experimental design showing incubations performed onboard (B). Stations are superimposed on sea surface chlorophyll-*a* concentration (mg m^−3^) obtained from 1/4° 10 day-binned COPERNICUS satellite data (https://data.marine.copernicus.eu/viewer) from 1 June to 1 July 2022. “T0” stands for time zero, “^13^C” and “^12^C” treatments indicate that the bottles were enriched with phytoplankton-derived labeled DOM previously produced in the lab (+DOM), “CTL” represents control incubations without added DOM and “^15^N_2_” shows the bottles that received enriched filtered seawater for N_2_ fixation measurements. Letters A–F refer to experimental replicates. DNA from incubations was used for *nifH* and *16S-rRNA* datasets.

This study was conducted during the NCD cruise (KM2206) between 4th June and 6th July 2022 onboard the R/V *Kilo Moana*. The cruise took place in the North Pacific Subtropical Gyre, west of the Hawaiian Islands between 15–30°N and 159–179°W (Fig. 1A). Seawater was collected from four stations (2, 4, 11, and 26; Fig. 1A) at 15 m depth and distributed into individual 4.5 l polycarbonate bottles (Nalgene, Rochester, NY, USA) to measure background conditions (time zero or “T0”), DOM uptake, and N_2_ fixation rates, and perform DNA-SIP analyses (Fig. 1B; Supplementary Information). Phytoplankton-derived DOM was extracted from cultures of *Synechococcus* sp. RCC2033 and *Thalassiosira pseudonana* previously grown in the lab following Kieft *et al.* ([23]; see Supplementary Information for more details on ^13^C/^12^C-labeled DOM production). This phytoplankton-derived DOM was added to the “DOM incubation bottles” to a final concentration of 8 μM C [~10% of background dissolved organic carbon (DOC) in surface waters of the North Pacific; [32, 33]; Fig. 1]. All incubations were performed on-deck incubators with flowing surface seawater for 24 h at *in situ* temperature in the dark (to reduce any osmotrophic signal from diazotrophic cyanobacteria and focus on that of NCDs). Subsamples for DOC, chromophoric and fluorescent DOM (CDOM and FDOM, respectively), dissolved inorganic nutrients (phosphate and nitrate, see below), and heterotrophic bacteria abundance were collected from all experimental bottles at the beginning of the experiment (T0), and after 18 h (T18) and 24 h (T24) of incubation (Fig. 1B; Supplementary Information). The volume remaining after sampling for DOC, CDOM, and FDOM was filtered either for DNA extractions (4 l onto 0.2 μm polysulfone membrane filters; Supor, Pall, Ann Arbor, MI, USA) or for particulate organic matter (POM; 4.4 l onto combusted GF/F filters; Whatman, Maidstone, UK) and POM isotopic enrichments analyses to measure N_2_ fixation and DOM uptake rates (see below; Supplementary Information).

### Water column measurements, nutrient, and dissolved organic matter analyses

A conductivity, temperature, and depth probe (CTD 9/11plus, Sea-Bird Scientific) mounted on a 24-Niskin bottle rosette sampler was used to measure hydrographic properties in the water column. Additional sensors included turbidity, beam attenuation, and chlorophyll-a fluorescence.

Samples for the measurement of nitrate and phosphate concentrations were obtained after filtration through GF/F filters in 20 ml Teflon vials and stored at −20°C until analysis (Supplementary Information). Samples for DOC, CDOM, and FDOM were collected by filtering through Milli-Q water- and sample-rinsed 0.45 μm GMF GD/X syringe filters (Whatman, Florham Park, New Jersey, USA) and stored in combusted (500°C, 4 h) 20 ml glass vials in the dark at 4°C prior to analysis (Supporting Information).

### Molecular characterization of phytoplankton-derived dissolved organic matter

Liquid chromatography coupled with high-resolution mass spectrometry was used simultaneously to detect and identify the metabolites in the phytoplankton-derived DOM extracts produced in the lab for onboard *in situ* incubations (see Supplementary Information). To analyze the (i) polar and (ii) apolar low-weight compounds, we injected 1 μl of each ^13^C-DOM and ^12^C-DOM extracts in triplicates and run them through a ZIC-HILIC column (150 × 2.1Cmm, 5 μm) and a Silica C18 column (100 ×  2.1 mm, 2.6 μm), respectively (see Supplementary Information for more details). The identity of selected compounds was confirmed with tandem mass spectrometry, and the MS/MS spectra were compared by spectral similarity search in Global natural product social networking (GNPS; see Supplementary Information).

The two data matrices were analyzed using the MetaboAnalyst 5.0 web tool [34], resulting in tables with ^12^C and ^13^C isotopic peaks from the same compounds as distinct variables (separated rows). Quantile normalization and normalization by sum methods were applied to the C18 and ZIC-HILIC datasets, respectively. Volcano plots were generated to identify the significant features discriminating between labeled and unlabeled DOM samples, using a fold change (FC) threshold of 2 and a *p* value threshold of 0.05 with False Discovery Rate correction.

### DNA stable-isotope probing

DNA-SIP experiments were performed by incubating natural planktonic communities with either heavily labeled (^13^C) or unlabeled (^12^C) DOM we had previously prepared from phytoplankton cultures in the lab [31, 35, 36] (Supplementary Information). The rationale behind using both heavy (^13^C) and light (^12^C) isotopes of the substrate of interest (here carbon contained in the DOM mixture) is to allow separation of the DNA of the substrate-incorporators by density differences (Supporting Information). After DNA extractions (see Supporting Information), heavy (high ^13^C-labelling) DNA was separated from light (low ^13^C-labelling and high ^12^C-labelling) DNA using a density gradient for both treatments, separating different molecular weight DNA fractions according to Neufeld *et al.* [31] (Supplementary Information). To verify the success of the DNA-SIP steps and determine the distribution of the DNA fractions after isopycnic separation, the abundance of 16S rRNA genes in each density fraction was quantified by quantitative polymerase chain reaction (qPCR). The qPCR assay was performed using the primers 968F and 1401R [37] as described in Cébron *et al.* [38] (Supporting Information). Based on the distribution of DNA and 16S rRNA copies along the density gradient and the comparison of ^13^C-enriched and ^12^C-enriched DNA samples (Fig. S1), we selected four consecutive DNA fractions here called heavy or “H”, medium or “M”, light or “L”, and super-light or “SL” for downstream sequencing analyses (*nifH* and 16S rRNA gene amplicon sequencing; see Supplementary Information).

### Statistical analyses

The integrated development environment for the statistical software R, RStudio (RRID: SCR_000432, Version 2023.12.1+402), was used to process and analyze the data and to generate graphs. All differences between treatments or stations for all parameters and other statistical patterns were evaluated by one-way analysis of variance (ANOVA), after checking data for normality and heterogeneity of variance (QQ plot, Shapiro–Wilk test, and Levene’s test). Significant differences in the relative abundance of *nifH* or 16S rRNA genes between the two treatments (^13^C vs. ^12^C) were tested using the Wilcoxon test. Statistical significance for all tests was set at a *P*-values <0.05 (95% confidence level).

## Results

### Biogeochemical and environmental patterns

Sea surface (<15 m) temperature and salinity differed significantly among stations (ANOVA, *P* < .0001; Fig. S2A and B), being highest at stations 26 and 11 (28°C and 35.3, respectively), and lowest at stations 4 and 2 (24°C and 34.9, respectively). Fluorescence and beam attenuation at the same depth were higher at stations 2 and 4 than at stations 11 and 26 (ANOVA, *P* < .0001; Fig. S2B and D; Supplementary Information). Nitrate and phosphate concentrations at 15 m ranged from 0.003 to 0.061 μM and from 0.007 to 0.060 μM, respectively, with the highest average concentrations observed at stations 26 and 2 and the lowest at station 4 (Fig. S2E and F). DOC concentrations at the same depth were lower at stations 2 and 4 (70 μM) than at the other two stations (84 μM; ANOVA; *P* < .001; Fig. S2G). The CDOM absorption coefficient at 325 nm (a_325_) and the humification and biological FDOM indices (HIX and BIX, respectively), indicated that DOM at station 2 (highest BIX, lowest a_325_) was fresher/more aliphatic than elsewhere. In contrast, DOM at station 4 (highest HIX, low BIX, high a_325_) displayed a more humic/aromatic character (Fig. S2I–K; [39, 40]). The average molecular weight of bulk DOM, depicted by the CDOM absorption spectral slope between 275 and 295 nm (S_275–295_), was higher at station 2 (lowest S_275–295_ values) and lower at the other stations (Fig. S2L). The high values of S_275–295_ and a_325_ at stations 4, 11, and 26 (Fig. S2K and L) indicated the dominance of low molecular weight and aromatic compounds, which potentially underwent photobleaching or other degradation processes [41].

### Molecular composition of phytoplankton-derived dissolved organic matter

Two data matrices were obtained for metabolite analyses of the phytoplankton-derived DOM extracts (^12^C and ^13^C) produced in the lab (Supplementary Information) and used as substrate in our onboard experiments. Together with the investigations of the isotopic peaks (Tables S1 and S2), volcano plots using both datasets indicated that most ^13^C isotopic peaks were more abundant in the ^13^C-DOM extract (right side), whereas the ^12^C isotopic peaks were more abundant in the ^12^C-DOM (left side; Fig. S3). Moreover several compounds, for example methyl-guanosine and valeryl-carnitine, showed high ^13^C atom enrichment (Fig. S4). Thus, these analyses confirmed that the composition of ^12^C- and ^13^C-DOM was very similar (Supporting Information), which is a prerequisite for DNA-SIP analysis [31]. After metabolite annotation, both datasets showed that the prominent chemical families corresponded to nitrogen-containing molecules such as amino acids (e.g. arginine, tryptophan), dipeptides (e.g. alanyl-leucine, glycyl-leucine), nucleosides (e.g. deoxyadenosine, deoxyguanosine), and carnitine derivatives (e.g. acetylcarnitine, propionylcarnitine; Tables S1 and S2). In addition, zwitterions such as dimethylsulfoniopropionate (DMSP) and choline were identified (Tables S1 and S2).

### Impact of phytoplankton-derived dissolved organic matter on N_2_ fixation

**Figure 2:**
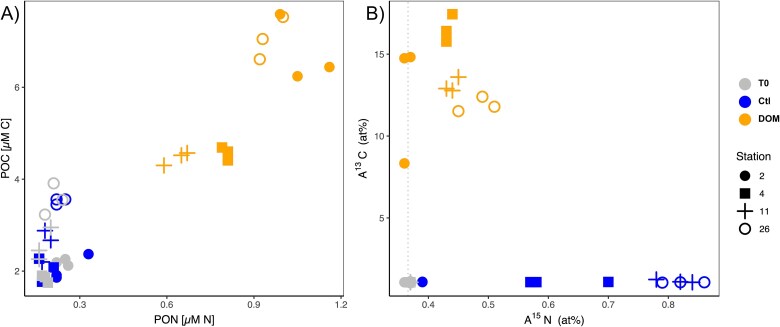
Bulk particulate organic matter (POC and PON; μM) (A) and atom ^15^N and ^13^C enrichments (at%) (B) from initial background samples (T0; grey), and after 24 h incubation of unamended (Ctl T24: Control; blue) and DOM-amended samples (DOM T24; orange) for each station (2, 4, 11, and 26, depicted by different shape).

Background (T0) concentrations of particulate organic carbon (POC) and nitrogen (PON; i.e. before DOM additions) were highest at stations 26 and 2, respectively (Fig. 2). POC and PON concentrations in the control incubations did not change after 24 h and were not significantly different from T0 values (*t*-test; *P* > .1; Fig. 2). Instead, a significant increase in both POC and PON was observed at all stations following DOM additions (*t*-test; *P* < .01; Fig. 2), with the highest and lowest POC and PON build-up measured at stations 2 and 11, respectively (Fig. 2A).

Given the potential inflation of bulk N_2_ fixation rates by background PON concentrations [42, 43], in this study we report the fractional ^15^N-enrichment of the particulate nitrogen (^15^N at %) which provides a more accurate measure of diazotrophic activity (N_2_ fixation). The ^15^N at% enrichment of bulk PON was higher in controls than in DOM-amended incubations at all stations (*t*-test, *P* < .05; Fig. 2B). Still, the ^15^N at% PON in DOM-amended samples was significantly higher than in T0 samples at all stations, except at station 2 (*t*-test, *P* > .1; Fig. 2B). The highest ^15^N at% PON values were observed at stations 26 and 11, which showed similar values (*t*-test, *P* > .05; Fig. 2B) regardless of whether the samples were DOM-amended or not (Fig. 2B). All DOM-amended samples showed significantly higher ^13^C at% POC enrichment than control and T0 samples (*t*-test, *P* < .05; Fig. 2B). The highest ^13^C at% POC was measured at station 4 and the lowest in one of the replicates at station 2, while all replicates at station 26 consistently showed the lowest values (Fig. 2B).

### Diazotroph community (*nifH* genes) response to dissolved organic matter additions

**Figure 3:**
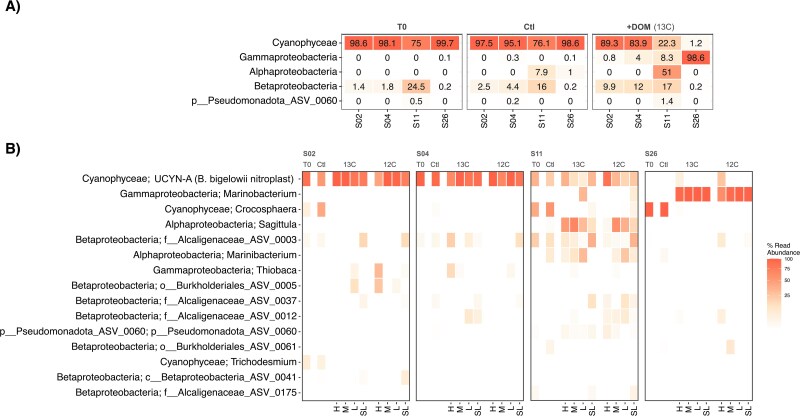
Heatmap showing the relative abundance of *nifH* gene reads in the diazotroph community for “T0” and post-incubation samples (unamended or “Ctl” and amended with either ^13^C- or ^12^C-DOM) at each station. The relative abundance of *nifH* reads is sorted by the most abundant class (A), and the top 15 most abundant taxa are further divided by genus (B) and into the different DNA density fractions (heavy “H”, medium “M”, light “L”, and superlight “SL”).

Phytoplankton-derived DOM additions caused a shift from diazotrophic cyanobacteria to NCDs (*nifH* genes) at stations where the abundance of the N_2_-fixing haptophyte was lower (stations 11 and 26). The T0 diazotroph community composition was heavily dominated by cyanobacteria over NCDs (93.1% over 6.9% of *nifH* reads) at all stations (Fig. 3A). Stations 2 and 4 showed the highest relative abundance of the *B. bigelowii* nitroplast (86.8% and 97%, respectively; Fig. 3B), previously referred to as UCYN-A. The relative abundance of the nitroplast at station 11 was similar to that of *Crocosphaera* (35.7% and 41.1% of *nifH* reads, respectively). In contrast, *Crocosphaera* dominated at station 26 (99.6%; Fig. 3B). *Trichodesmium nifH* reads were found at low relative abundance at stations 2 and 26 (6.5% and 0.1%, respectively; Fig. 3B). The few NCDs at T0 were mainly assigned to the Alcaligenaceae family (betaproteobacteria; Fig. 3A), particularly at station 11 (Fig. 3B).

After 24 h of incubation, the relative abundance of the nitroplast in control incubations decreased by 35.6% and 12.5% at stations 2 and 11, respectively, but remained almost constant at station 4 (Fig. 3B). The initially high relative abundance of *Crocosphaera* at station 26 remained relatively constant during the incubation but increased to 36.1% and 7.0% at stations 2 and 11, respectively (Fig. 3B). The relative abundance of *Trichodesmium* at station 2 also decreased during the control incubations (Fig. 3B). Conversely, an alphaproteobacterium of the genus *Marinibacterium* had higher relative abundance (20.5% and 1.0% at station 11 and 26, respectively) in control incubations than at T0 (Fig. 3B).

Phytoplankton-derived DOM additions increased the relative abundance of two alpha- and one gammaproteobacteria NCDs annotated as *Sagittula*, *Marinibacterium* and *Marinobacterium,* respectively, at stations 26 and 11 (Fig. 3B). The relative abundance of the nitroplast in DOM-amended incubations decreased by 17.5 and 14.3% at stations 4 and 11, respectively, and only slightly (2.5%) at station 2. No *Trichodesmium nifH* reads were detected in the DOM-amended samples, and *Crocosphaera* was only detected at low abundance at stations 11 and 26 (0.4% and 0.1% of total *nifH* reads, respectively; Fig. 3).

**Figure 4:**
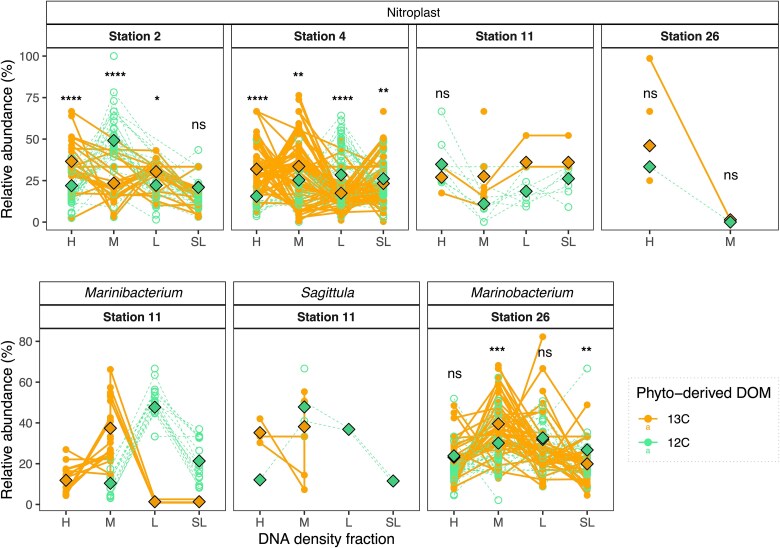
*nifH* gene relative abundance of enriched (higher in ^13^C fractions) amplicon sequence variant (ASVs; assigned to genus) across DOM treatments (^13^C-labeled: Filled dot and solid line, and ^12^C-labeled; open dot and dashed line) and density fractions (heavy or “H”, medium or “M”, light or “L”, and super-light or “SL”). Each point represents the average of the three experimental replicates. Different lines within each panel indicate different ASVs assigned to the same genus. Diamond dots show the average of all ASVs in each fraction and treatment. Significant differences in relative abundance between DOM treatments were tested with the Kruskal-Wallis test and shown as significant codes (^****^P < .0001; ^***^P < .001; ^**^P < .01; ^*^P < .1; ns; not significant).

Beyond bulk changes in the relative abundance of the *nifH* gene between control or DOM-amended incubations (Fig. 3), DNA-SIP analyses allowed us to identify which diazotrophic taxa incorporated organic carbon from the added DOM mixture (Fig. 4). We examined changes in the relative abundance of *nifH* genes in the H, M, L, and SL DNA density fractions comparing ^13^C- and ^12^C-DOM amended samples (Fig. 4). The nitroplast (Fig. 4A), the alphaproteobacteria *Sagittula* and *Marinibacterium* (Fig. 4B and C), and the gammaproteobacterium *Marinobacterium* (Fig. 4D) were the main diazotrophs showing evidence of DOM incorporation (Fig. 4). The relative abundance of the nitroplast was higher in the H ^13^C DNA fraction than in the H ^12^C DNA fraction at stations 2 and 4 (Kruskal–Wallis test; *P* < .0001; Fig. 4). The *nifH* genes of the alphaproteobacteria *Marinibacterium* and *Sagittula* were not detected in H ^12^C DNA fractions, while their relative abundance represented 11.8% and 35.2% of the *nifH* relative abundance in the H ^13^C DNA fraction (Fig. 4B and C). The relative abundance of the gammaproteobacterium *Marinobacterium* was slightly higher in the H ^13^C DNA fraction than in the H ^12^C DNA fraction (Kruskal–Wallis test; *P* = .6102; Fig. 4D). Moreover, the *nifH* relative abundance of the nitroplast, *Marinibacterium* and *Marinobacterium* in M ^13^C DNA fractions were also 1.32, 3.59, and 1.31 times higher than in M ^12^C DNA fractions treatment at stations 4, 11 and 26, respectively (Kruskal–Wallis test; *P* < .01; Fig. 4).

### Overall prokaryote community (16S ribosomal ribonucleic acid genes) response to dissolved organic matter additions

**Figure 5:**
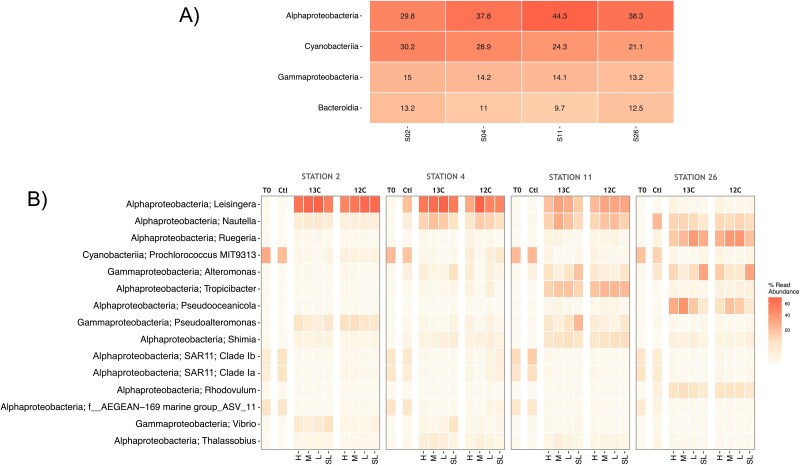
Heatmap showing the relative abundance of 16S rRNA gene amplicon reads assigned to the four most abundant classes at “T0” (A) and to the 15 most abundant ASVs shared between “T0” and post-incubation samples (unamended or “Ctl” and amended with ^13^C- or ^12^C-DOM) (B) for each station. For each taxon shown in B, the relative abundance of 16S rRNA gene reads is further divided into the different DNA density fractions (heavy or “H”, medium or “M”, light or “L”, and superlight or “SL”).

The 16S rRNA gene amplicon sequencing revealed that phytoplankton-derived DOM additions significantly increased the relative abundance of several alpha- and gammaproteobacteria groups, while alphaproteobacteria showed higher osmotrophic capacities. Groups of alphaproteobacteria dominated at T0 at all stations (29.8%–44.3%), followed by the non-diazotrophic cyanobacterium *Prochlorococcus* (21.1%–30.2%; Fig. 5A). Gammaproteobacteria and Bacteroidia represented 13.2%–15% and 9.7%–13.2% of the total prokaryotic community, respectively, showing less variability among stations (Fig. 5A). In general, the abundance of heterotrophic bacteria estimated by flow cytometry (cells ml^−1^) did not vary during control incubations (*t*-test; *P* > .1; Fig. S5A), except at station 2 where their abundance increased significantly over the incubation period (*t*-test; *P* = 2 × 10^−5^). In control incubations, the initially dominant alphaproteobacteria belonging to SAR11 clades Ia and Ib, and the marine group AEGEAN-169 were present together with *Prochlorococcus,* but their relative abundance did not change by more than 6% as compared to T0 (Fig. 5B).

**Figure 6:**
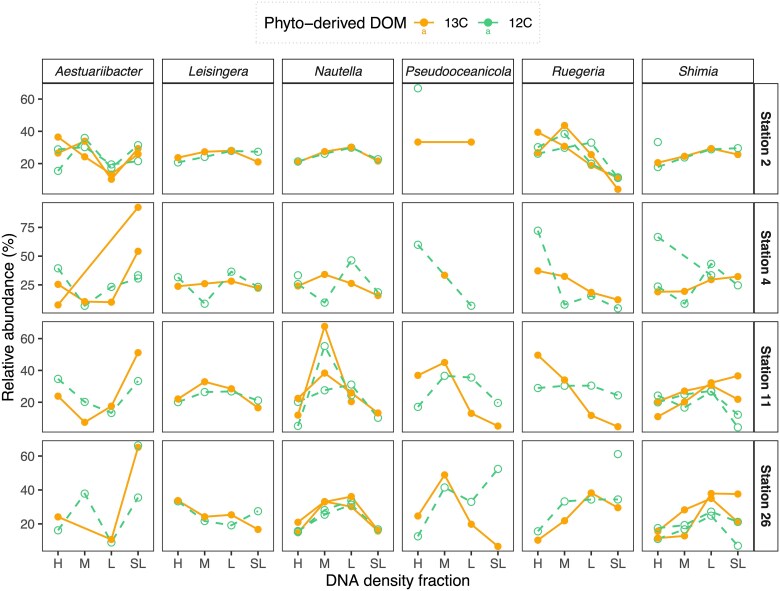
Relative abundance (16S rRNA gene) profiles of enriched (higher in ^13^C fractions) ASVs (assigned to genus) across DOM treatments (^13^C-labeled: Filled dot and solid line, and ^12^C-unlabelled; open dot and dashed line) and density fractions (heavy or “H”, medium or “M”, light or “L”, and super-light or “SL”). Each point represents the average of the three experimental replicates. Different lines within each panel indicate different ASVs assigned to the same genus.

The abundance (cells ml^−1^) of heterotrophic bacteria increased at all stations following DOM additions (*P* < .1; Fig. S5B), being mostly representatives of alpha- and gammaproteobacteria groups (Fig. 5B). However, most groups showed similar relative abundances between ^13^C- and ^12^C-DOM incubations when contrasting different DNA density fractions (e.g. H or M; Fig. 6), suggesting no DOC incorporation. This was the case for most gammaproteobacteria, including *Pseudoalteromonas* and *Alteromonas* (Fig. 5B). Similarly, the relative abundance of some alphaproteobacteria such as *Shimia,* which increased significantly after DOM additions (Fig. 5B), was not higher in the H ^13^C DNA than in the H ^12^C DNA fractions (Fig. 6). In contrast, we observed a significant increase in the relative abundance of the alphaproteobacteria *Leisingera* (stations 2 and 11), *Nautella* (stations 11 and 26), *Pseudooceaonicola* (stations 11 and 26), and of *Ruegeria* (stations 2 and 11) in the heavier (H and M) ^13^C DNA fractions as compared to the corresponding ^12^C DNA fractions (Fig. 6).

To evaluate the competition and partitioning of DOM between diazotrophic and non-diazotrophic bacteria, we did a co-occurrence network analysis (see Supplementary Information; Fig. S6) using both the *nifH* and 16S rRNA gene reads from the different DNA-SIP fractions. These networks showed positive connections between alphaproteobacteria (diazotrophic or not), while negative connections were observed between gamma- and alphaproteobacteria, and between diazotrophic and non-diazotrophic gammaproteobacteria (Fig. S6). Negative relationships between alphaproteobacteria taxa were only observed between T0 abundant oligotrophic groups such as Clade Ia and all alphaproteobacteria taxa after DOM addition, and between *Leisingera* and *Ruegeria* with the nitroplast (Fig. S6).

## Discussion

Phytoplankton-derived DOM additions to surface diazotroph communities revealed that both the *B. bigelowii* nitroplast and diverse NCDs were able to take up DOM. However, the response of diazotrophs to DOM additions varied largely among groups and between stations, influenced by temperature, nutrient concentrations, DOM composition and differences in the *in situ* community structure (Fig. S6).

### Dissolved organic matter uptake by non-cyanobacterial diazotrophs

The alphaproteobacteria *Sagittula* and *Marinibacterium*, and the gammaproteobacterium *Marinobacterium* (see Supplementary Information for *nifH* sequence homology) assimilated phytoplankton-derived DOM at the westernmost and warm waters (26.31°C–27.82°C; Fig. S2) stations 11 and 26 (Fig. 4). Previous studies have reported gammaproteobacteria as the dominant NCD group in open waters of the Pacific and Atlantic Oceans [5, 44, 45], with their *nifH* gene counts or relative abundances positively correlating with nutrients and DOM availability or primary productivity [13, 14, 46, 47]. In contrast, *nifH* reads of the alphaproteobacterium *Sagittula* negatively correlate with nitrate and phosphate concentrations in regions such as the Eastern Indian Ocean [48]. Consistent with this, the detection of *Sagittula nifH* gene reads at station 11 coincided with low phosphate concentrations (Fig. S2F). Indeed, the genome of *Sagittula* shows diverse metabolic pathways to obtain dissolved organic phosphorus, including phosphonates and phosphoanhydrides [49], which may allow this genus to thrive in phosphate-poor waters when other resources such as DOM are not limiting. While *Sagittula* has been suggested as an important N_2_ fixer worldwide (e.g. [49, 50]), *Marinibacterium* and *Marinobacterium* have rarely been reported from open ocean samples [51, 52]. *Marinibacterium* MAGs suggest that their metabolism is versatile, including the ability for photoheterotrophy (anoxygenic photosystem II; 52) or to use methanol as a carbon and energy source (methanol dehydrogenase, *XoxF*; 51). However, our knowledge of their activity and involvement in biogeochemical cycles is still limited.

Based on available reference genomes, both *Sagittula* and *Marinobacterium* are flexible in substrate utilization (e.g. sugars, amino acids, and peptides) and energy acquisition mechanisms, including the degradation of aromatic hydrocarbon compounds [49, 53]. The ^13^C-DOM substrate used in our incubations contained several compounds (i.e. amino acids and nucleosides; Tables S1 and S2) that can be utilized by both *Sagittula* and *Marinobacterium*. For example, *Marinobacterium* can synthesize glycine betaine from choline [53]. Glycine betaine is an important osmoprotectant, as well as DMSP and carnitine, which were present in our DOM mixture (Tables S1 and S2). These ubiquitous metabolites and their derivatives, such as DMS, are well known to serve as energy and/or nutrient sources for most prokaryotes [54, 55], including *Sagittula* [49] and even eukaryotes such as marine diatoms [56, 57]. *Sagittula* and *Marinobacterium* may have benefitted similarly from these widespread marine metabolites during our study. Still, *Sagittula* genes encode for the uptake of a wider variety of substrates than *Marinobacterium* species, such as lipopolysaccharides, lipoproteins, tungstate, and thiamine [49], which might explain their prevalence at station 11 where the lowest biomass was observed (Fig. 2). In contrast, *Marinobacterium* is less metabolically versatile but still had higher relative abundances than *Crocosphaera* upon phytoplankton-derived DOM additions at station 26, suggesting an efficient uptake of the added DOM allowing for rapid growth (Fig. 3). This contrasts with previous studies showing DOM uptake by *Crocosphaera* [13, 21]. However, in those studies, photosynthesis was not limited (day/night cycle incubations, while our incubations were 24 h in the dark), and the amended DOM consisted mainly of carbohydrates which were not detected in our ^13^C-DOM mixture (Tables S1 and S2). In addition, the growth rates of different microorganisms may affect the amount of ^13^C incorporated into DNA at a given time, as DNA needs to be replicated to be detected as a ^13^C-DOM signal. Therefore, even if slow-growing microorganisms take up significant amounts of ^13^C-DOM, the incorporation of isotopic labels in their DNA can be low, while a longer incubation to counteract this problem might introduce bias as cross-feeding events [58].

Overall, our results indicate that different NCD groups grew on DON-rich DOM (Tables S1 and S2), allowing them to outcompete other diazotrophs but did not favor bulk N_2_ fixation. NCDs are considered facultative N_2_ fixers as they show broad flexibility in their nitrogen metabolism [7]. Yet, N_2_ fixation rates were detectable after DOM additions and ^15^N at% PON values were significantly higher than at T0 at stations where *Sagittula*, *Marinibacterium,* and *Marinobacterium* were present (Fig. 2B). These NCDs groups were virtually absent at T0 at stations 11 and 26 but increased their relative abundance upon the addition of phytoplankton-derived DOM. This observation could be due to the low lability of the background DOM at T0 (Fig. S2I–L) or to the dominance of better-adapted photoautotrophic species such as *Crocosphaera.* Our results suggest that *Sagittula*, *Marinobacterium,* and *Marinobacterium* can contribute to DOM uptake and compete with other prokaryotes even when nitrogen metabolites are available.

### Dissolved organic matter uptake in the N_2_-fixing haptophyte nitroplast

The *B. bigelowii* nitroplast assimilated DOM at the eastern and cooler waters (24.28°C–25.94°C; Fig. S2) stations 2 and 4 (Figs 3 and 4). These stations differed significantly from each other in the background chemical composition and DOM lability (Fig. S2). At station 2, DOM was fresher and had a higher molecular weight (higher BIX and lower S_275–295_ values characteristic of newly released DOM by either bloom crash or zooplankton grazing [59, 60]; Fig. S2J and L) than at station 4, which was more chemically complex and refractory (e.g. higher HIX, a_325_ and S_275–295_ values; Fig. S2I, K, and L). Furthermore, nitrate and phosphate concentrations were the lowest at station 4 (Fig. S2). These differences in the background availability of DOM and nutrients partly explain why higher ^13^C-DOM assimilation was observed at station 4 than at station 2 (Fig. 4). This indicates that DOM uptake is a beneficial trait for the coccolithophore *B. bigelowii* under dark and low nutrient availability conditions. At station 2, *B. bigelowii* might have assimilated less DOM or even used some of the more labile background DOM present in ambient waters, resulting in lower incorporation of ^13^C-DOM and an isotopic dilution of the ^13^C signal in DNA extracts. Still, ^13^C-DOM additions at station 2 induced a 15.45-fold increase in the relative abundance (bulk DNA) of nitroplast *nifH* reads (*t*-test; *P* = .075; Fig. 3), suggesting that compounds other than organic carbon in the DOM mixture (e.g. nitrogen-rich amino acids or nucleosides; Tables S1 and S2) may have stimulated their growth.

**Figure 7:**
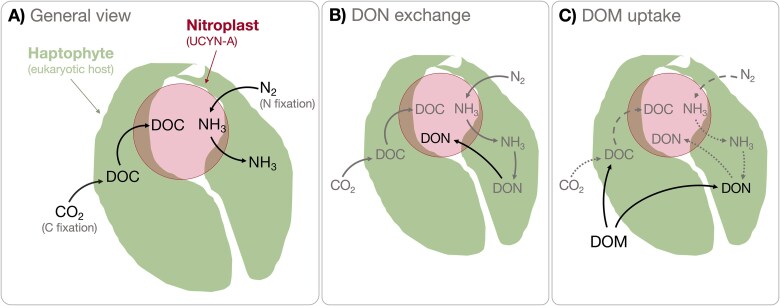
Schematic figure illustrating the known (A and B) and potential (C) carbon and nitrogen acquisition and exchange pathways between the environment and the haptophyte *Braarudosphaera bigelowii* and its nitroplast. Dotted lines indicate when the process might be decreased due to a new type of uptake or reallocation of resources.

Mills *et al.* [61] found that *B. bigelowii* does not take up nitrate and assimilates only small amounts of ammonium, suggesting that its nitrogen requirements are met mainly by N_2_ fixation by the nitroplast (62; Fig. 7A). However, the nitroplast may fail to meet the nitrogen demand of *B. bigelowii* under conditions where the ratio of carbon fixation/nitrogen transfer or *B. bigelowii*/nitroplast size is unbalanced [61, 62]. In these cases, the *B. bigelowii*/nitroplast is likely to rely on other reactive nitrogen sources such as dissolved organic nitrogen (DON) or bacterial phagotrophy [63]. The nitroplast lacks the genetic machinery to produce some key organic nitrogen compounds, such as amino acids and nucleotides. Still, the nitroplast can incorporate such compounds from the algae via specific amino acid or purine transporters [64] (Fig. 7B). This suggest an intricate exchange of nitrogenous metabolites, with N_2_ being fixed into ammonia in the nitroplast and then transferred to *B. bigelowii* [3]. In return, the host metabolizes ammonium into organic nitrogen, which is then transferred to the nitroplast (Fig. 7B).

N_2_ fixation (presented as ^15^N at% enrichment) was undetectable or lower than controls after DOM additions at stations 2 and 4 (Fig. 2), suggesting that DOM inhibited N_2_ fixation. Using DOM as a nitrogen source instead of relying on its nitroplast may reduce the overall energy requirements of *B. bigelowii* (Fig. 7C). Flexibility in substrate use and resource allocation may be a key trait of *B. bigelowii*, explaining its ability to thrive from tropical to polar oceans [65, 66], and to survive in turbid upwelling waters where diazotrophic cyanobacteria are uncompetitive [9, 67]. Indeed, although light appears to be a critical factor controlling the metabolism of the *B. bigelowii* symbiosis, the carbon fixed by the algae might be crucial in regulating N_2_ fixation in the nitroplast [68]. The dark incubations used in our experiments may have triggered an osmotrophic response of *B. bigelowii* and subsequent transfer of ^13^C-DOM to the nitroplast (Fig. 7C) at stations where it was initially abundant. These results call for a review of the role of the *B. bigelowii*/nitroplast ocean DOM cycling in the ocean.

### Competition between diazotrophic and non-diazotrophic bacteria for dissolved organic matter

Bulk POC and PON concentrations increased after DOM additions to dark incubations (Fig. 2), suggesting that microbial growth was DOM-limited without light. The POC ^13^C at% enrichment confirmed substrate incorporation by the bulk planktonic community at all stations, while the PON ^15^N at% enrichment was consistently higher in control than in DOM-amended incubations (Fig. 2). This indicates a potential suppression of N_2_ fixation by DOM, e.g. by DON metabolites, which constituted most of the molecules detected in our DOM mixture (Tables S1 and S2), or the faster uptake of DOM by microbes other than diazotrophs, limiting resources for N_2_ fixation (Fig. 5). A combination of these scenarios is likely, and therefore the increase of ^13^C at% enrichment in bulk POC arguably includes the signal from both diazotrophic and non-diazotrophic microbes (Fig. 2, Fig. 5).

To further understand the competition and partitioning of DOM between diazotrophic and non-diazotrophic bacteria, we evaluated the differences in 16S rRNA gene reads in the different DNA fractions as we did for the *nifH* genes (Figs 5 and 6). Although the non-diazotrophic prokaryotic community was similar between stations at T0 (Fig. 5A), and in contrast to the diazotrophic community (Fig. 3A), we did not observe an analogous prokaryote response to DOM additions at the different stations (Fig. 5B). Therefore, factors other than the metabolic capabilities of the non-diazotrophic prokaryotes such as nutrient availability or competition with other planktonic groups may have shaped the DOM uptake at the different stations. In general, the relative abundance of non-diazotrophic prokaryotes increased significantly after DOM additions, especially alphaproteobacteria (Figs 5B and S6). Notwithstanding, only a few groups showed ^13^C-DOM assimilation and their response varied spatially, indicating different use of the DOM and intraspecies competition at the different stations (Figs 5B, 6, and S6). For example, the alphaproteobacteria *Leisingera* and *Nautella* increased their relative abundance after DOM addition at most stations, including station 4. Still, they only showed evidence of DOM uptake at stations 2 and 11, and 11 and 26, respectively (Fig. 6). At station 4, where the background DOM was refractory and nutrients were scarce (Fig. S2), DOM additions induced a high ^13^C at% enrichment of POC (Fig. 2B), suggesting that the *B. bigelowii* nitroplast may be a significant contributor to DOM assimilation at this station. Conversely, the other prokaryotes that increased in relative abundances after DOM addition (bulk DNA) at station 4 may have used the phytoplankton-derived DOM as a source other than organic carbon such as nutrients (e.g. nitrogen-containing metabolites; Tables S1 and S2). This could explain the uncoupling between increasing bacterial growth and DOM incorporation, especially at stations 4 and 26 (Figs 2A, 5, and S5A). At station 26, the increase in relative abundance of the non-diazotrophic alphaproteobacterium *Ruegeria* and of the two gammaproteobacteria, *Alteromonas* and *Pseudoalteromonas*, in response to DOM additions was also uncoupled from DOM uptake (Fig. 6). Again, these species could have benefited from DOM additions for different purposes such as deriving their sulfur requirements from DMSP, as observed in several *Ruegeria* species [69]. In contrast, another study conducted during our cruise analyzing particle-associated NCDs found that gammaproteobacteria were the most abundant groups [70], suggesting that POM is a more suitable carbon source for gammaproteobacteria NCDs than DOM.

Our results suggest that the variability in ambient nutrients, DOM, and community structure between stations drives contrasting responses to DOM additions between alpha- and gammaproteobacteria. The negative relationship between these two classes following DOM additions is supported by co-occurrence network analyses (Fig. S6). Moreover, this analysis showed mostly positive connections between alphaproteobacteria taxa (diazotrophic or not), revealing very different ecologies within this class and a diverse and shared exploitation of DOM enabling different planktonic groups to benefit from the same substrate. The weak negative relationship between *Leisingera* and the nitroplast at stations 2 and 4 (Fig. S6) indicates some competition between the two groups for the added substrate. Longer incubations and nutrient addition experiments will help disentangle the competition of different bacterial groups for DOM. Such studies emerge as a priority in the increasingly warmer and nutrient-starved subtropical gyres, where competition for DOM substrates between microbial species may influence carbon cycling and contribution of DOM to the biological carbon pump.

## Supplementary Material

SI_NCDC_final_ycaf061(1)

## Data Availability

All the data generated by this experiment and supporting results and figures are available in the Figshare repository at figshare/b45dd99d490237ff23d1. *nifH* and 16S amplicon sequencing data are deposited in NCBI BioSample database and are accessible through the BioProject accession number PRJNA1099264 (https://www.ncbi.nlm.nih.gov/sra/PRJNA1099264). Additional Supporting Information can be found in the online version of this article. The results of the GNPS molecular networking with metabolite identification can be found for C18 analysis at: https://gnps.ucsd.edu/ProteoSAFe/status.jsp?task=b3e0015f19c544c1a155ee99415d6d2c. and for the ZIC-HILIC analysis at: https://gnps.ucsd.edu/ProteoSAFe/status.jsp?task=32e8c60be66b4bf9a690f611c7d52cb6. The LCMS raw spectra (.mzXML, .RAW), samples list and the compounds lists have been deposited in the MassIVE metabolomics repository (C18 dataset MassIVE MSV000096448: ftp://MSV000096448@massive.ucsd.edu; Zic-Hilic Dataset MassIVE MSV000096449: ftp://MSV000096449@massive.ucsd.edu).
