# Supplementary material for: Organic metabolite uptake by diazotrophs in the North Pacific Ocean
Source: ISME Commun. 2025 May 5;5(1):ycaf061. doi: 10.1093/ismeco/ycaf061 (PMC12064561; doi:10.1093/ismeco/ycaf061)
Supplement: SI_NCDC_final_ycaf061(1) [file si_ncdc_final_ycaf061(1).docx]

Organic Metabolite Uptake by Diazotrophs in the North Pacific Ocean

^1,2,3^Alba Filella, ^4^Aurélie Cébron, ^5^Benoît Paix, ^6,7^Marine Vallet, ^1^Pauline Martinot, ^1^Léa Guyomarch, ^1^Catherine Guigue, ^1^Marc Tedetti, ^1^Olivier Grosso, ^8^Kendra A. Turk-Kubo, ^9^Lasse Riemann, ^1,2,10,*^Mar Benavides

^1^Aix Marseille Univ, Université de Toulon, CNRS, IRD, MIO UM 110, 13288, Marseille, France

^2^Turing Center for Living Systems, Aix-Marseille University, 13009 Marseille, France

^3^Department of Molecular and Cellular Biology, The University of Arizona, Tucson, AZ, USA

^4^Université de Lorraine, CNRS, LIEC, Nancy, France

^5^UMR CARRTEL, INRAE - Université Savoie Mont-Blanc, Thonon-les-Bains, France

^6^Max Planck Fellow Group Plankton Community Interactions, Max Planck Institute for Chemical Ecology Jena, Germany

^7^Institute for Inorganic and Analytical Chemistry, Friedrich Schiller University of Jena, Jena, Germany

^8^Ocean Sciences Department, University of California, Santa Cruz, Santa Cruz, CA, USA

^9^Department of Biology, University of Copenhagen, Denmark

^10^National Oceanography Centre, European Way, SO14 3ZH Southampton, UK

*Corresponding author: [mar.benavides@noc.ac.uk](mailto:mar.benavides@ird.fr)

# Supplementary Information

## Supplementary Methods

### Experimental design and sampling procedure

Seawater was collected at night using Teflon pump and tubing (Saint-Gobain Performance Plastics, Saint-Quentin-Fallavier, France), in 40 l acid-cleaned carboys and then distributed into 21 individual 4.5 l polycarbonate bottles (Fig. S1B). Twelve bottles were kept as controls (no DOM added), from which six were immediately processed as ‘T0’ background conditions and the other six used as incubation ‘controls’ to measure bulk N_2_ fixation rates and to assess the community changes over the incubation period (Fig. 1B; see below). The remaining twelve bottles were amended with phytoplankton-derived DOM for DNA-SIP analysis (^12^C- and ^13^C-DOM, three replicates each), and to measure DOM uptake rates and asses the effect of DOM in bulk N_2_ fixation rates (all six bottles received ^13^C-DOM) (Fig. 1B). At T0, the total concentration of DOC in the DOM incubation bottles (i.e., background seawater DOM plus the added phytoplankton-derived DOM) ranged between 79.3 and 88.5 µM. For N_2_ fixation measurements, three unamended controls and three ^13^C-DOM-amended bottles, received 10% ^15^N_2_ enriched seawater (Fig. 1B). The remaining nine incubation bottles (DNA-SIP bottles, DOM uptake incubations and unamended controls; Fig. 1B) were mixed with 10% 0.2 µm filtered seawater to maintain the same dilution factor as the N_2_ fixation rate bottles in all treatments.

### Water column measurements, nutrient and DOM analysis

Inorganic nutrient concentrations were determined using a segmented flow autoanalyzer (AAIII HR, Seal Analytical) according to Aminot and Kérouel [1]. The detection limits and analytical precision were 0.050 and 0.003 μM for nitrate and 0.020 and 0.001 μM for phosphate. Chlorophyll-*a* was measured using a Turner Fluorometer TD-700 (Turner Designs, Inc., San Jose, CA) [2].

DOC samples were spiked with 20 µl of sulfuric acid before storage. DOC was measured on three replicates by high-temperature catalytic oxidation using a Shimadzu TOC-V Total Carbon Analyzer [3]. The accuracy and system blanks of the instrument were determined by the analysis of Deep Atlantic Water and low carbon water reference standards (D. Hansell, Rosenstiel School of Marine and Atmospheric Science, Miami, USA). The nominal precision of the analysis procedure was within 2%. CDOM absorbance spectra were generated in single beam mode at between 200 and 800 nm with a wavelength interval of 1 nm, a slit width of 1 nm, and a scan speed of 230 nm min^-1^. The precision of the absorbance measurements was > 97% over the range of 220-700 nm. The absorbance values were corrected for blank and baseline drift and then converted into CDOM absorption coefficients (a(λ) in m^-1^) [4]. The spectral slope between 275 and 295 nm (S_275-295_ in nm^-1^) was estimated from the natural log-transformed absorption spectra following Helms et al. [5]. This region of the spectra is susceptible to changes in the molecular weight of CDOM, with higher slopes denoting lower average molecular weight [5, 6]. For FDOM analyses, excitation-emission matrices (EEMs) were generated over an excitation wavelength (λ_Ex_) range of 200–500 nm at 5 nm intervals and an emission wavelength (λ_Em_) range of 280-550 nm at 2 nm intervals, a scan speed of 1 200 nm min^-1^, slit widths of 5 nm, and a PMT voltage of 700 V [7, 8]. The EEMs were corrected for inner filtering effects using absorption measurements and the formula described in Ohno [9], blank-corrected by subtracting the ultrapure water EEMs and converted into quinine sulfate units (QSU) [10, 11]. Because the variability of the Raman scatter peak of ultra-pure water during the whole study period was very low, normalization of the fluorescence intensities was not applied. We also determined two fluorescence indices: the humidification index (HIX), considered as an indicator of the degree of humification of the DOM [9, 12], and the biological index (BIX), considered to relate to the level of freshness of the DOM [13].

### Production of phytoplankton-derived DOM

Cultures of *Thalassiosira pseudonana* (AC589; Algobank–Caen culture collection, Paris, France) and *Synechococcus* sp. (RCC2033; Roscoff Culture Collection, Roscoff, France) were grown in Guillard′s F/2 (Merck Life Science, Saint-Quentin-Fallavier, France) and PCR-S11 [14] media, respectively. The media were sparged with helium overnight, amended with ^12^C- or ^13^C-labeled 1 mM NaHCO_3_ (Merck Life Science, Saint-Quentin-Fallavier, France), and autoclaved before inoculating the cultures. Equivolume (2 l) mixtures of the two phytoplankton cultures at mid exponential phase were filtered onto several 47 mm combusted glass fiber filters (GF/F; Whatman, Maidstone, UK), using precombusted glassware. Filters were then rinsed with 500 ml of 0.2 µm filtered seawater with low DOC content (~60 µM C), and measurements were done before and after rinsing to ensure that the cells on the filters were free of residual DOC (e.g., EDTA from culture media). The filter content was then resuspended into 200 ml Milli-Q water and then sonicated for one hour, frozen at -80°C, thawed at room temperature, and sonicated again for another hour before the last filtration through combusted GF/F filters to recover the intracellular DOM. Subsamples from each DOM extract (^13^C and ^12^C) used as substrates for the DNA-SIP incubations were taken to do metabolomics (see below) and analyze the stoichiometric composition of DOC and total dissolved nitrogen and phosphorus content and the carbon isotope labeling by elemental analyzer isotope ratio mass spectrometry (EA-IRMS) (see below).

### Molecular characterization of phytoplankton-derived DOM

The analytical separation and quantification of the metabolites present in our phytoplankton-derived DOM extracts were completed using a Dionex Ultimate 3000 system (Thermo Scientific, Dreieich, Germany) connected to a Q-Exactive Plus Orbitrap mass spectrometer (Thermo Scientific, Dreieich, Germany). The columns were kept at 25 °C and the injection volume was 1 μl. Electrospray ionization was performed in positive mode ionization with the following parameters: capillary temperature, 380 °C; spray voltage, 3000 V; sheath gas flow, 60 arbitrary units; and aux gas flow, 20 arbitrary units.

The UHPLC column was a SeQuant ZIC-HILIC column (2.1 x 150 mm, 5 μm) coupled with a SeQuant ZIC-HILIC guard column (2.1 x 20 mm, 5 μm) (Merck, Darmstadt, Germany). For the separation of osmolytes with ZIC-HILIC chromatography, the gradient elution was performed using isocratic elution of 100% solvent B (90% acetonitrile with 10% 1 mmol of aqueous ammonium acetate LC-MS grade, LGC Promochem, Wesel, Germany) for 1 min, followed by a linear gradient from 100% to 20% solvent B for 5.5 min and a linear gradient from 20% to 100% for 0.6 min, ending with an isocratic equilibration at 100% solvent B for 2.9 min. The total run time was 10 min and flow rate was set to 0.6 ml min-1. For the C18 analysis, the metabolite separation was performed in a 12 min gradient, starting with 100% of the aqueous phase (2% acetonitrile and 0.1% formic acid in water) and increasing with the acetonitrile phase (0.1% formic acid in acetonitrile) within 8 min until reaching 100%. This was held for 3 min before switching back to 100% of the water phase and equilibration for 1 min. The flow rate was 0.4 ml min^-1^.

Mass spectrometry was conducted in positive ion mode with a scan range of *m*/*z* 75 to 1125 at a peak resolution of 280 000 for the MS1 acquisition. For MS2 acquisition using a ddMS experiment, MS2 spectra were acquired at a peak resolution of 17 500. AGC target was set to 1 × 10^5^ and maximum ion time was set to 50 ms. The MS/MS spectra of precursor ions within an isolation window of *m*/*z* 1.5 at a peak resolution of 17 500 (NCE 20, 30, 40). Apex trigger was set to 5 to 10 s and dynamic exclusion was set to 10 s. The MS2 parameters employed were set to the ones suggested for GNPS analysis [15].

Mass tolerance for mass spectrometry identification was 5 ppm, minimum mass spectrometry peak intensity was 2 × 10^5^, and intensity tolerance for isotope search was 30%. The relative standard deviation value was set to 50%. To analyze labeled compounds, the maximum exchange rate was set to 25 and the Source efficiency to 100%.

Mass spectrometry chromatograms were visualized with the software Xcalibur (Thermo Fisher Scientific), and raw data processing, peak deconvolution, and metabolite annotation were done in Compound Discoverer 3.3 using the stable isotope labeling workflow (Thermo Fisher Scientific).

The lists containing the selected labeled compounds were exported as ‘.xlsx’ files and the mass were searched in public mass lists (LipidsMaps, Natural Products Atlas, Thermo libraries). The raw LC-MS data were converted into an open-source file format (‘.mzXML’ and ‘.mgf’) using the software ProteoWizard and further evaluated in GNPS and Sirius. GNPS is an analysis method to provide chemical insight into MS2-data and gives structural context between possibly related compounds of the metabolome. It works as a tool for the annotation of marine compounds and serves as a repository for untargeted MS2-data. With the molecular networking tool of the GNPS website, a data table of every metabolite captured from the previously recorded MS data was created and interlinked into a network connecting similar structured components while pointing out labeled compounds. A molecular network was created using the online workflow (<https://ccms-ucsd.github.io/GNPSDocumentation>) on the GNPS website (<http://gnps.ucsd.edu>).

The data was filtered by removing all MS/MS fragment ions within +/- 17 Da of the precursor *m*/*z*. MS/MS spectra were window-filtered by choosing only the top 6 fragment ions in the +/- 50 Da window throughout the spectrum.

The precursor ion mass tolerance was set to 0.02 Da and a MS/MS fragment ion tolerance of 0.02 Da. A network was then created where edges were filtered to have a cosine score above 0.7 and more than 4 matched peaks. Further, edges between two nodes were kept in the network if and only if each of the nodes appeared in each other's respective top 10 most similar nodes. Finally, the maximum size of a molecular family was set to 50, and the lowest scoring edges were removed from molecular families until the molecular family size was below this threshold. The spectra in the network were then searched against GNPS' spectral libraries. The library spectra were filtered in the same manner as the input data. All matches kept between network spectra and library spectra were required to have a score above 0.7 and at least 4 matched peaks. The molecular network for the dataset analyzed on the C18 column in positive mode can be found here: <https://gnps.ucsd.edu/ProteoSAFe/status.jsp?task=b3e0015f19c544c1a155ee99415d6d2c> . The molecular network for the dataset analyzed on the ZIC-HILIC column in positive mode can be found here: <https://gnps.ucsd.edu/ProteoSAFe/status.jsp?task=32e8c60be66b4bf9a690f611c7d52cb6> .

### DNA-SIP

The principle of DNA-SIP stands on the idea that if the organic carbon from DOM is incorporated by a given organism into its DNA, that process is equally going to occur at both treatments independently (^13^C- or ^12^C-DOM). The final DNA concentration after incubation will be the same between treatments, but not the molecular weight of the DNA. The DNA will be heavily labeled (^13^C) at the ^13^C-DOM treatment while it will keep mostly ^12^C-labeled at the ^12^C-DOM treatment. After DNA extractions, if the carbon has been assimilated, the ^13^C-DNA will be shifted towards heavier density fractions than the ^12^C-DNA (see below).

DNA samples were obtained before and after incubations of natural communities with DOM (see above; Fig. 1A) by filtering 4 l subsamples onto 0.2 μm polysulfone membrane filters (Supor, Pall, Ann Arbor, MI, USA). Filters were stored in sterile 2 ml bead beater tubes containing 0.1 mm glass beads (BioSpec, Bartlesville, OK, USA) and immediately flash-frozen in liquid nitrogen and stored at −80°C. In the laboratory, cell breakage was promoted by three liquid nitrogen flash-freezing and bead-beating cycles. DNA was then extracted using the mini-Plant Kit according to manufacturer guidelines (Qiagen Sciences, Germantown, Maryland, USA) with a 4 h proteinase K digestion step [16]. DNA concentrations were quantified using the PicoGreen dsDNA Quantification Kit (Invitrogen, Thermo Fisher Scientific).

For DNA-SIP analyses, Neufeld et al. [17] recommend adding 5 µg of DNA to the density gradient, however, due to the low DNA concentrations obtained here, we used the entire amount of DNA recovered from each sample, ranging between 130 and 1230 ng. DNA was added to Quick-Seal polyallomer tubes (13 × 51 mm, 5.1 ml, Beckman Coulter, Lyon, France), along with a gradient buffer (0.1 M Tris–HCl, 0.1 M KCl, 1 mM EDTA) mixed with a CsCl solution to a final buoyant density of 1.88505 g ml^−1^. The tubes were ultracentrifuged at 176 985 × g (VTI 65.2 rotor, Beckman, Roissy, France) at 20ºC for 40 h. Following centrifugation, 12 DNA fractions of ∼ 400 μl were recovered from each tube. The buoyant density value of each fraction was measured with a refractometer (VWR, Rosny-sous-Bois, France), the average difference in buoyant density between successive fractions being ∼ 0.0005 g ml^−1^. DNA was recovered from each fraction by overnight precipitation with 20 μg glycogen (MP Biomedicals) and 700 μl of polyethylene glycol solution (30% PEG 6 000, 1.6 M NaCl), followed by centrifugation for 45 min at 13 000 g. DNA was rinsed with 70% ethanol, air-dried and resuspended in 30 μl of molecular-grade water.

For the qPCR assay, we use a 20 μl reaction mixture consisting of 10 μl of 10 μl iQ SYBR green SuperMix (Bio-Rad), 0.8 μl of each primer (10 μM), 0.4 μl bovine serum albumin (3%), 0.2 μl dimethyl sulfoxide, 0.08 μl T4gp32 (MP Biomedicals, France) and 1 μl DNA template (DNA fraction samples or standards from 10^8^ to 10^2^ 16S rRNA copies µl^-1^). Quantifications were performed using a CFX96 Real-Time PCR detection system (Bio-Rad, Marne-la-Coquette, France) with the following conditions: initial denaturation at 95ºC for 5 min, followed by 39 cycles of denaturation at 95ºC for 30 s, annealing at 56ºC for 20 s, elongation at 72ºC for 30 s and measurement of SYBR Green signal intensities at 82ºC for 5 s.

The four fractions (H, M, L and SL) had the following densities: 1.40036 g ml^−1^,1.39985 g ml^−1^,1.39925 g ml^−1^ and 1.39871 g ml^−1^, respectively.

### nifH and 16S rRNA gene amplicon sequencing and bioinformatics

Triplicate DNA-SIP fractions of each isotopic concentration (H, M, L or SL) and bulk (‘T0’ and control) samples were run through nested PCR reactions using degenerate *nifH* primers [18, 19]. The second-round primers were modified with adapters for Illumina library preparation using a Doppio thermocycler (VWR, Radnor, PA). The PCR mix was composed of 5 μl of 5× MyTaq red PCR buffer (Bioline, Meridian Bioscience, United Kingdom), 1.25 μl 25 mM MgCl_2_, 0.5 μl 20 μM forward and reverse primers each, 0.25 μl GoTaq® G2 Flexi DNA Polymerase (Promega, Madison, WI, USA) and 5 μl DNA extract (1 μl in the second round). The reaction volume was adjusted to 25 μl with PCR grade water, and PCR conditions were as described in Benavides et al. [20]. Triplicate PCR products were pooled and purified using the PCR clean-up for PCR products (Machery-Nagel, Düren, Germany). Amplicon quality was checked via Agilent DNA ScreenTape (Agilent Technologies, Les Ulis, France). The purified products were indexed using the Nextera XT Index Kit from Illumina (San Diego, CA, USA), which attaches dual indices and Illumina sequencing adapters. Indexed products were purified (AMPure XP beads, Agencourt, Beckman Coulter Genomics, Lyon, France), quantified (Qubit dsDNA assay kits, Thermo Fisher Scientific), adjusted to equimolar concentrations, and pooled for multiplex sequencing on Illumina MiSeq 2 × 300 bp paired-end sequencing platform (Macrogen, Amsterdam, The Netherlands).

Paired-end demultiplexed raw sequences were processed into amplicon sequencing variants (ASVs) using DADA2 [21] in RStudio Version 4.2.2 (ref for that R version). The parameters used for filtering and trimming reads were as follows: truncLen = c(210,170), maxN = 0, maxEE = c(2,2), truncQ = 2, m.phix = TRUE, trimLeft = c(17,17). Denoised reads were merged and chimeric sequence removed using the "mergePairs" and "removeBimeraDenovo" functions respectively. Only sequences with a length of 325 to 328 bp were retained. Following the NifMAP pipeline [22], the retained ASVs were translated into amino acid sequences using Framebot [23] and filtered for homologous genes. No homologous genes were found. After taxonomy assignment against a *nifH* DADA2 database (Moynihan & Furbo Reeder 2023; http://doi.org/10.5281/zenodo.7996213), all sequences with bootstrap <80% at the order level were removed and not analyzed further. Furthermore, ASVs detected only in one of the two isotope treatments (^12^C and ^13^C) across density fractions were removed from the enrichment analysis to exclude possible artifacts of the methodology itself.

16S rRNA gene sequencing was done using 515F-806R primers with Illumina-specific adapters [24, 25], generated following standard protocols of amplicon library preparation (16S Metagenomic Sequencing Library Preparation, Illumina, Part #15044223 Rev.B), as recommended by the Earth Microbiome project (<https://earthmicrobiome.org/>). Briefly, PCR products were generated with 50 µl of total reaction volume containing 22 µl PCR grade water, 10 µl MyTaq Polymerase, 1 µl of each forward and reverse primer (10 µM), 2.5 µl MgCl_2_ (25 mM), 1µl BSA (10 mg ml^-1^) and 12 µl DNA template previously normalized to 2 ng µl^-1^. All PCR products were cleaned using the MP Biomedicals™ Geneclean™ Turbo Kit and normalized to 25 ng µl^-1^. Normalized PCR products were submitted to Azenta Genwitz for subsequent Library preparation and Amplicon sequencing. 16S rRNA gene amplicon reads were generated using 300-bp paired-end sequencing using an Illumina NextSeq sequencer. Raw sequences were imported and processed in R (v4.2.2) and RStudio (v2023.06.0 + 421). We used the DADA2 (v1.26.0) pipeline to process raw sequences into amplicon sequence variants (ASVs). Denoised reads were merged using the function mergePairs(), and chimeric contigs were removed removeBimeraDenovo(). Taxonomic ranks for ASVs generated from 16S sequences were assigned using the Silva 138.1 prokaryotic SSU database for DADA2 (McLaren & Callahan 2021; https://zenodo.org/records/4587955).

For simplicity and considering that diazotrophs usually represent <1/6 of the prokaryotic community in the ocean, we consider 16S rRNA sequences as the non-diazotrophic prokaryotic community.

For the co-occurrence and network analysis (Fig. S6) we used the co_occurrence_network function from the phylosmith package (<https://schuyler-smith.github.io/phylosmith>) in R which works with the *phyloseq* object previously created by combining both the *nifH* and 16S rRNA gene abundance data using DADA2 (see above).

### Particulate organic matter and isotope measurements

Particulate organic nitrogen (PON) and carbon (POC) concentrations and ^13^C/^12^C and ^15^N/^14^N isotope ratios were determined by EA-IRMS (INTEGRA 2, Sercon Ltd, Crewe, UK). After incubation, ~4.4 l of the incubated seawater was filtered onto pre-combusted (450°C, 4 h) GF/Fs, dried at 60°C for 24 h, and stored at room temperature until analysis. Before and after the analysis of every ten samples, the instrument was calibrated using IAEA-600 reference material (caffeine), and the quantification limits were calculated as ten times the standard deviation of GF/F filter blank analyses. The quantification limit was ~ 5 μg for nitrogen and ~ 15 μg for carbon. All ^15^N and ^13^C atom % values were above the linearity limit calculated as a function of increasing mass of particulate organic nitrogen and carbon, respectively. N_2_ fixation rates were calculated as described in Montoya et al. [26], and the ^15^N isotope enrichment was obtained as the difference between natural abundances in ‘background’ and treatment samples after incubation. The ^15^N/^14^N ratio from the dissolved N_2_ pool was measured by membrane inlet mass spectrometry [27] by sampling the filtrate from the POM samples avoiding any bubbles in 12 ml gas-tight exetainers, which were kept at 4°C until analysis. For all rate measurements the standard propagation of errors was calculated via partial derivatives [28] to determine the minimum quantifiable rate (MQR), obtaining values ranging from 0.32 to 5.91 nmol N l^-1^ d^-1^ for stations 2 and 26, respectively. Bulk DOM uptake rates were similarly calculated from ^13^C isotope enrichment differences in POC (^13^C at%) between natural abundances and treatment samples after incubation, and the ^13^C source enrichment was determined from background and amended DOC volume and concentrations.

### Heterotrophic bacteria abundance

A volume of 1.98 ml was subsampled from each incubation bottle and experiment time point into cryotubes, spiked with 20 µl of a mix of glutaraldehyde (25%) and pluronic acid (10%) at 100:10 dilution, fixed for 15 min at room temperature, and finally flash-frozen in liquid nitrogen and stored at −80°C until analysis. In the laboratory, samples were stained with 2 µl SYBR Green I (Thermo Fisher Scientific, Dardilly, France) at 1:10 of the stock solution (10 000x, Thermo Fisher Scientific, Eugene, Oregon, USA). Heterotrophic bacteria (HB) were enumerated using a CytoFLEX flow cytometer (Beckman Coulter, Villepinte, France) at the PRECYM Platform (https://precym.mio.osupytheas.fr/). Trucount^TM^ beads (BD Biosciences, Paris, France) were used to determine the analyzed volume, and fluoresbrite 2 µm latex beads (Polysciences, Inc., Warrington, PA, USA) were added as internal size standards. Samples were run at low speed (10–30 μl min^−1^), and HB were identified in a plot of side scatter (SSC) versus green fluorescence.

##

## Supplementary Results and Discussion

### Biogeochemical and environmental patterns

The deep chlorophyll maximum was deeper (~140 m) and less intense (fluorescence < 0.5 RFU) at stations 2 and 26 than at the other stations (Fig. S2C). At station 11, the deep chlorophyll maximum coincided with the highest peak of the beam attenuation at about 90 m (Fig. S2C-D). At station 4, the fluorescence maximum was similar in magnitude (fluorescence > 0.75 RFU) to that at station 11 but deeper, located at about 120 m (Fig. S2C-D). Fluorescence-derived Chlorophyll-*a* concentrations were 1.3 times higher at stations 2 and 4 (~0.070 µg l^-1^) than at the other two stations (~0.056 µg l^-1^) (Fig. S2H; ANOVA; p < 0.001). These profiles indicated that the water column at station 26 followed by station 2 was more mixed than at the other stations where stronger stratification was observed (Fig. S2B).

### Molecular composition of phytoplankton-derived DOM

Diverse compounds were found to be ^13^C-labeled with an enrichment from 1 to 3 ^13^C atoms (e.g., propionylcarnitine, arginyl-valine) or more (e.g., up to six ^13^C atoms for seryl-leucine and alanyl-leucine; and up to ten ^13^C atoms for phenylalanine-proline) (Tables S1, S2). Specifically, the methyl-guanosine and valeryl-carnitine characterized, labeled with up to 6 and 2 ^13^C atoms, respectively, showed the highest fold change (FC) values of the dataset (Log_2_ (FC) < -3.5 for ^12^C isotopes and Log_2_ (FC) > 3 for all ^13^C isotopes) (Fig. S4).

### NCDs nifH sequence homology

The *nifH* reads annotated to *Sagittula* had 100% nucleotide similarity to the NCD *Sagittula castanea*, previously isolated from the Eastern Tropical South Pacific [29]. Most *nifH* sequences annotated as *Marinibacterium* showed 91.98% amino acid similarity to *Marinibacterium anthonyi*, isolated from North Atlantic coastal waters [30] and less abundant *Marinibacterium* sequences showed 100% amino acid similarity to those from the Tara Oceans circumnavigation dataset (MAG_00020; 31). The *Marinobacterium nifH* reads had high protein sequence similarity to isolates of *M. litorale*, *M. zhoushanense* and *M. ramblicola* species (Query Cover = 99% and Per. Indent = 100%; 32–34).

## Supplementary Tables

**Table S1**. Putative annotation of the unlabeled and labeled compounds from the phytoplankton-derived DOM mix given to the natural diazotrophic communities from the C18 LC-ESI-(+)-MS/MS analyses. All compounds were detected with [M+H]^+^ adducts. ^a^Green and red colors indicate ^12^C and ^13^C isotopes respectively.

| Putative annotation | Isotope^a^ | m/z | RT (sec) | Formula | MS/MS fragmentation patterns |
| --- | --- | --- | --- | --- | --- |
| Acetylcarnitine | ^12^C [M+H]^+^ | 204.1231 | 49.8 | C9H17NO4 | 145.0496 [C6H9O4]+ ; 85.02843 [C4H5O2]+ ; 60.08117 [C3H10N]+ |
|  | ^13^C [M+1+H]^+^ | 205.1267 | 49.8 | C9H17NO4 | 159.1126 ; 146.0529 ; 118.0862 ; 85.0284 ; 60.0448 |
|  | ^13^C [M+2+H]^+^ | 206.1297 | 48.6 | C9H17NO4 | 147.0561 ; 146.0529 ; 87.0350 ; 86.0317 ; 85.0284 ; 61.0845 |
|  | ^13^C [M+3+H]^+^ | 207.1329 | 46.8 | C9H17NO4 | 148.0595 ; 147.0562 ; 146.0528 ; 88.0383 ; 84.0350 ; 86.0317 ; 85.0284 ; 62.0872 ; 61.08 ; 60.0811 |
|  | ^13^C [M+6+H]^+^ | 210.1436 | 49.8 | C9H17NO4 | 150.0665 ; 149.0631 ; 89.0420 ; 88.0386 ; 63.0913 ; 62.0872 ; 61.0483 |
| Alanylleucine  (Ala-Leu) | ^12^C [M+H]^+^ | 203.139 | 107.94 | C9H18N2O3 | 157.1334 ; 132.102 ; 130.0863 [C6H11NO2]+ ; 86.0964 [C5H11N]+ ; 84.0809 [C5H9N]+ ; 84.0444 [C4H5NO]+ |
|  | ^12^C [M+H]^+^ | 203.1388 | 73.32 | C9H18N2O3 | 157.1334 ; 132.102 ; 130.0863 [C6H11NO2]+ ; 86.0964 [C5H11N]+ ; 84.0809 [C5H9N]+ ; 84.0444 [C4H5NO]+ |
|  | ^13^C [M+1+H]^+^ | 204.1421 | 104.4 | C9H18N2O3 | 158.1368 ; 133.1053 ; 132.10287.0998 ; 86.0964 |
|  | ^13^C [M+2+H]^+^ | 205.145 | 103.2 | C9H18N2O3 | 159.14 ; 134.1086 ; 133.1052 ; 88.1030 ; 87.0997 |
|  | ^13^C [M+3+H]^+^ | 206.1488 | 103.2 | C9H18N2O3 | 160.1436 ; 135.112 ; 134.1087 ; 89.1064 ; 88.1031 ; 87.0998 |
|  | ^13^C [M+6+H]^+^ | 209.1588 | 105 | C9H18N2O3 | 163.1539 ; 162.1502 ; 137.1189 ; 136.1155 ; 90.1098 ; 89.1065 |
| Arginine | ^12^C [M+H]^+^ | 175.1188 | 36.9 | C6H14N4O2 | 158.0923 [C6H11N3O2]+ ; 157.1079 [C6H12N4O]+ ; 133.0976 [C5H12N2O2]+ ; 116.0706 [C5H9NO2]+ ; 115.0867 [C5H10N2O]+ ; 98.0599 [C5H7NO]+ ; 97.0761 [C5H8N2]+ ; 70.0654 [C4H7N]+ ; 71.0492 [C4H6O]+ ; 130.0976 [C5H11N3O]+ ; 112.0870 [C5H9N3]+ ; 74.0967 [C4H11N]+ ; 60.0560 [CH5N3]+ ; 72.0811 [C4H9N]+ |
|  | ^13^C [M+2+H]^+^ | 177.1257 | 41.94 | C6H14N4O2 | 131.1007 ; 118.0774 ; 117.0738 ; 80.9479 ; 71.0687 ; 72.07208 ; 61.0594 ; 60.0560 |
|  | ^13^C [M+4+H]^+^ | 179.1323 | 40.2 | C6H14N4O2 | 134.1111 ; 133.1077 ; 120.084 ; 119.0807 ; 82.9450 ; 80.9479 ; 73.0537 ; 75.0720 ; 61.05933 ; 60.05585 |
| Asparagylisoleucine  (Asn-Ile) | ^12^C [M+H]^+^ | 246.1461 | 115.8 | C10H19N3O4 | 229.1183 [C10H15N2O4]+ ; 212.0918 [C10H14NO4]+ ; 187.1078 [C8H15N2O3]+ ; 166.0862 [C9H12NO2]+ ; 141.1023 [C7H13N2O]+ ; 132.102 [C6H14NO2]+ ; 87.0553 [C3H7N2O)+ ; 86.0964 [C5H12N]+ |
|  | ^13^C [M+3+H]^+^ | 249.154 | 125.4 | C10H19N3O4 | 231.1246 ; 215.1017 ; 214.0952 ; 203.1304 ; 168.0927 ; 143.1089 ; 135.1119 ; 134.1086 ; 133.1053 ; 89.04256 ; 88.1030 ; 87.0997 |
| Aspartyl-Isoleucine  (Asp-Ile) | ^12^C [M+H]^+^ | 247.1284 | 138.42 | C10H18N2O5 | 229.1177 [C10H16N2O4]+ ; 201.1233 [C9H16N2O3]+ ; 187.1076 [C8H14N2O3]+ ; 212.0914 [C10H13NO4]+ ; 132.1018 [C6H13NO2]+ ; 86.0963 [C5H11N]+ |
| Alanyl-Proline  (Ala-Pro) | ^12^C [M+H]^+^ | 187.1077 | 46.26 | C8H14N2O3 | 116.0706 [C5H9NO2]+ ; 86.0965 [C5H11N]+ ; 84.0444 [C4H5NO]+ ; 72.0811 [C4H9N]+ ; 84.0808 [C5H9N]+ ; 70.0654 [C4H7N]+ |
| Deoxyadenosine | ^12^C [M+H]^+^ | 252.1084 | 67.56 | C10H13N5O3 | 136.0617 [C5H5N5]+ ; 117.0545 [C5H8O3]+ ; 99.0439 [C5H6O2]+ ; 73.0286 [C3H4O2]+ ; 71.0494 [C4H6O]+ |
|  | ^13^C [M+1+H]^+^ | 253.1122 | 67.62 | C10H13N5O3 | 137.0653 ; 136.0619 ; 118.0579 ; 117.0547 ; 73.0286 |
|  | ^13^C [M+4+H]^+^ | 256.1223 | 79.8 | C10H13N5O3 | 139.072 ; 138.0687 ; 137.0653 ; 119.0614 ; 101.0508 ; 74.032 |
| Dipeptide derivative | ^12^C [M+H]^+^ | 261.1438 | 127.68 | C11H20N2O5 | 243.1336 [C11H18N2O4]+ ; 225.1231 [C11H16N2O3]+ ; 215.1390 [C10H18N2O3]+ ; 197.1285 [C10H16N2O2]+ ; 132.1019 [C6H13NO2]+ ; 86.0964 [C5H11N]+ |
|  | ^13^C [M+1+H]^+^ | 262.146 | 136.18 | C11H20N2O5 | isotopic patterns of M+1+H^+^ |
| Glutaminylisoleucine | ^12^C [M+H]^+^ | 260.1599 | 112.56 | C11H21N3O4 | 243.1338 [C11H18N2O4]+ ; 242.1498 [C11H19N3O3]+ ; 189.1236 [C8H16N2O3]+ ; 225.1235 [C11H16N2O3]+ ; 197.1285 [C10H16N2O2]+ ; 198.1132 [C10H15NO3]+ ; 169.1333 [C9H16N2O]+ ; 86.0964 [C5H11N]+ ; 132.1020 [C6H13NO2]+ |
|  | ^13^C [M+1+H]^+^ | 261.1638 | 107.4 | C11H21N3O4 | 244.1362 ; 243.1529 ; 198.1318 ; 133.1054 ; 132.1019 ; 87.09961 ; 86.0963 ; 84.0443 ; 83.0603 |
|  | ^13^C [M+6+H]^+^ | 266.1804 | 108 | C11H21N3O4 | 249.1539 ; 248.17 ; 203.1487 ; 202.146 ; 137.0461 ; 89.1065 |
| Glycyleucine | ^12^C [M+H]^+^ | 189.1238 | 80.4 | C8H16N2O3 | 86.0964 [C5H12N]+ |
|  | ^13^C [M+1+H]^+^ | 190.1267 | 70.8 | C8H16N2O3 | 87.0997 ; 86.0964 |
|  | ^13^C [M+5+H]^+^ | 194.399 | 72.6 | C8H16N2O3 | 91.1131 ; 90.110 ; 89.1064 ; 88.1031 |
| Guanosine derivative | ^12^C [M+H]^+^ | 225.1536 | 109.2 | C10H11N5O5 | 152.0564 [C5H6N5O]+ ; 147.0649 (?) ; 136.0618 (?) |
|  | ^13^C [M+1+H]^+^ | 283.0863 | 102 | C10H11N5O5 | 153.06 ; 152.0566 |
| Hexadecenoic acid  (derivative) | ^12^C [M+H-H2O]^+^ | 251.2001 | 404.46 | C16H28O3 | 251.2004 [C16H26O2]+ ; 167.1060 [C10H14O2]+ ; 223.2054 [C15H26O]+ ; 233.1898 [C16H24O]+ ; 153.0909 [C9H12O2]+ ; 125.0962 [C8H12O]+ |
| Tryptophan | ^12^C [M+H]^+^ | 205.097 | 155.82 | C11H12N2O2 | 188.0704 ; 170.0599 [C11H7NO]+ ; 160.0759 [C10H9NO]+ ; 146.0601 [C9H7NO]+ ; 144.0809 [C10H9N]+ ; 118.0651 [C8H7N]+ |
|  | ^13^C [M+3+H]^+^ | 208.0993 | 156.6 | C11H12N2O2 | 191.0807 ; 173.0701 ; 162.1023 ; 149.0701 ; 148.0668 |
|  | ^13^C [M+6+H]^+^ | 211.1166 | 157.2 | C11H12N2O2 | 194.0908 ; 176.0802 ; 165.1116 ; 164.1085 ; 152.0802 ; 151.0768 ; 150.0735 |
| Isoleucyl-Threonine | ^12^C [M+H]^+^ | 233.1493 | 52.26 | C10H20N2O4 | 215.1389 [C10H18N2O3]+ ; 120.0655 [C4H9NO3]+ ; 104.0706 [C4H9NO2]+ ; 86.0964 [C5H11N]+ |
|  | ^13^C [M+6+H]^+^ | 233.1493 | 52.26 | C10H20N2O4 | 221.1581 ; 90.10972 ; 89.1064 ; 88.1030 |
| Isoleucylproline | ^12^C [M+H]^+^ | 229.1544 | 149.22 | C11H20N2O3 | 116.0706 [C5H9NO2]+ ; 86.0964 [C5H11N]+ ; 72.0812 [C4H9N]+ ; 70.0653 [C4H7N]+ ; 69.0701 [C5H8]+ |
|  | ^13^C [M+1+H]^+^ | 230.1577 | 149.23 | C11H20N2O3 | 117.0739 ; 116.0706 ; 87.09975 ; 86.0964 ; 71.0687 ; 70.0654 |
| Leucylisoleucine | ^12^C [M+H]^+^ | 245.1856 | 170.04 | C12H24N2O3 | 199.1806 [C11H22N2O]+ ; 132.1018 [C6H13NO2]+ ; 129.1023 [C6H12N2O]+ ; 86.0963 [C5H11N]+ ; 84.0807 [C5H9N]+ ; 70.0653 [C4H7N]+ ; 69.0701 [C5H8]+ |
| Lysyl-isoleucine | ^12^C [M+H]^+^ | 260.1964 | 54.06 | C12H25N3O3 | 243.1702 [C12H22N2O3]+ ; 242.1862 [C12H23N3O2]+ ; 147.1129 [C6H14N2O2]+ ; 132.1020 [C6H13NO2]+ ; 86.0965 [C5H11N]+ ; 130.0864 [C6H11NO2]+ ; 129.1023 [C6H12N2O]+ ; 118.0866 [C5H11NO2]+ ; 100.1119 [C6H13N]+ ; 112.0757 [C6H9NO]+ ; 102.0914 [C5H11NO]+ ; 85.0649 [C5H8O]+ ; 84.080 [C5H9N]+ |
| Methyladenosine | ^12^C [M+H]^+^ | 282.119 | 107.28 | C11H15N5O4 | 136.0619 [C5H5N5]+ ; 147.0653 [C6H10O4]+ ; 152.0566 [C5H5N5O]+ ; 129.0546 [C6H8O3]+ ; 115.0392 [C5H6O3]+ |
|  | ^13^C [M+2+H]^+^ | 284.1253 | 103.32 | C11H15N5O4 | 138.0685 ; 137.0651 ; 136.0617 ; 154.0633 ; 153.0598 ; 152.0565 |
|  | ^13^C [M+3+H]^+^ | 285.1284 | 102.6 | C11H15N5O4 | 139.0719 ; 138.0685 ; 137.0651 ; 155.0665 ; 154.0632 ; 153.0598 |
|  | ^13^C [M+4+H]^+^ | 286.1327 | 104.4 | C11H15N5O4 | 140.0757 ; 139.0722 ; 138.0688 ; 156.0701 ; 155.0669 ; 154.0635 |
|  | ^13^C [M+7+H]^+^ | 289.1428 | 103.8 | C11H15N5O4 | 141.079 ;140.0756 ; 139.0722 ; 157.0736 ; 156.0703 ; 155.0669 |
| Phenylalanylleucine  (Phe-Leu) | ^12^C [M+H]^+^ | 279.17 | 3.14 | C15H22N2O3 | 262.1438 [C15H20NO3]+ ; 233.1643 [C14H21N2O]+ ; 132.1018 [C6H14NO2]+ ; 120.0807 [C8H10N]+ ; 86.0963 [C5H12N]+ |
|  | ^13^C [M+1+H]^+^ | 280.1739 | 2.98 | C15H22N2O3 | 234.1676 ; 133.1054 ; 132.1022 ; 121.0843 ; 120.0809 ; 87.04 |
|  | ^13^C [M+3+H]^+^ | 282.1801 | 3.17 | C15H22N2O3 | 236.1738 ; 134.1088 ; 133.0857 ; 123.0908 ; 122.0874 ; 121.0841 ; 89.0596 |
|  | ^13^C [M+4+H]^+^ | 283.1829 | 3.16 | C15H22N2O3 | 237.1788 ; 133.0857 ; 124.0942 ; 123.0908 ; 122.0875 ; 121.0841 ; 89.0596 |
|  | ^13^C [M+5+H]^+^ | 284.187 | 3.16 | C15H22N2O3 | 238.1815 ; 125.0946 ; 124.0942 ; 123.0908 ; 88.103 ; 87.0997 |
| Phenylalanylthreonine (Phe-Thr) | ^12^C [M+H]^+^ | 267.1336 | 1.56 | C13H18N2O4 | 249.1228 [C13H17N2O3]+ ; 152.0564 (?) ; 120.0806 [C8H10N]+ |
|  | ^13^C [M+1+H]^+^ | 268.1375 | 1.58 | C13H18N2O4 | 250.126 ; 153.0601 ; 152.0566 ; 121.0842 ; 120.0808 |
|  | ^13^C [M+3+H]^+^ | 270.1443 | 1.57 | C13H18N2O4 | 252.134 ; 154.0637 ; 153.0602 ; 123.091 ; 122.0876 |
|  | ^13^C [M+4+H]^+^ | 271.1462 | 1.55 | C13H18N2O4 | 253.1361 ; 155.0669 ; 154.0634 ; 153.06 ; 124.0942; 123.0908 ; 122.0875 |
|  | ^13^C [M+5+H]^+^ | 272.1512 | 1.57 | C13H18N2O4 | 254.1405 ; 155.0668 ; 154.0634 ; 125.0976 ; 124.0943 ; 123.0909 |
|  | ^13^C [M+7+H]^+^ | 274.1576 | 1.52 | C13H18N2O4 | 256.1467 ; 157.0733 ; 156.0703 ; 155.0667 ; 126.101 ; 125.0976 ; 124.0943 |
| Phenylalanineproline  (Phe-Pro) | ^12^C [M+H]^+^ | 263.1386 | 162 | C14H18N2O3 | 246.1125 [C14H16NO3]+ ; 217.1361 [C13H17N2O]+ ; 120.0807 [C8H10N]+; 116.0706 [C5H10NO2]+ ; 104.0527 ; 70.0653 [C4H8N]+ |
|  | ^13^C [M+1+H]^+^ | 264.142 | 163.8 | C14H18N2O3 | 247.1834 ; 218.1406 ; 121.0841 ; 120.0808 ; 117.0739 ; 116.0706 ; 70.0653 |
|  | ^13^C [M+3+H]^+^ | 266.149 | 165 | C14H18N2O3 | 249.1215 ; 123.0908 ; 122.0875 ; 121.0842 ; 120.0809 ; 118.0773 ; 117.074 ; 116.0707 ; 72.0721 ; 71.0687 ; 70.0654 |
|  | ^13^C [M+4+H]^+^ | 267.1521 | 166.2 | C14H18N2O3 | 124.0943 ; 123.0908 ; 122.0875 ; 121.0842 ; 119.0807 ; 118.0773 ; 117.0739 ; 116.0706 ; 73.0752 ; 72.0720 ; 71.0687 ; 70.0654 |
|  | ^13^C [M+5+H]^+^ | 268.1552 | 164.4 | C14H18N2O3 | isotopic pattern of M+5+H+ |
|  | ^13^C [M+8+H]^+^ | 271.1654 | 165.6 | C14H18N2O4 | 247.8704 ; 126.1009 ; 125.0976 ; 124.0943 ; 120.0842 ; 119.0807 ; 118.0773 ; 86.09644 ; 72.08101 |
|  | ^13^C [M+10+H]^+^ | 273.1814 | 161.4 | C14H18N2O4 | 126.1009 ; 125.0975 ; 124.0943 ; 86.0964 |
| Phenylalanylvaline  (Phe-Val) | ^12^C [M+H]^+^ | 265.1543 | 160.2 | C14H20N2O3 | 219.1489 [C13H18N2O]+ ; 120.0807 [C8H9N]+ ; 103.0541 [C8H6]+ ; 147.1129 [C6H14N2O2]+ ; 130.0862 [C6H11NO2]+ ; 129.1022 [C6H12N2O]+ ; 118.0862 [C5H11NO2]+ |
|  | ^13^C [M+4+H]^+^ | 269.1672 | 161.4 | C14H20N2O3 | 223.1625 ; 124.0942 ; 123.0908 ; 122.0875 ; 121.0841 |
| Prolylproline  (Pro-Pro) | ^12^C [M+H]^+^ | 213.1232 | 48.36 | C10H16N2O3 | 167.1176 [C9H14N2O]+ ; 138.0543 [C7H7NO2]+ ; 135.1171 [C10H14]+ ; 116.0705 [C5H9NO2]+ ; 86.0964 [C5H11N]+ ; 70.0653 [C4H7N]+ ; 84.0807 [C5H9N]+ |
| Propionylcarnitine | ^12^C [M+H]^+^ | 218.1386 | 68.82 | C10H19NO4 | 159.0651 [C7H10O4]+ ; 144.1022 [C7H13NO2]+ ; 85.0285 [C4H4O2]+ ; 60.0812 [C3H9N]+ ; 57.0338 [C3H4O]+ |
|  | ^13^C [M+1+H]^+^ | 219.1416 | 69.858 | C10H19NO4 | 160.0685 [C7H11O4]+ ; 86.03178 [C4H5O2]+ ; 85.0284 [C4H5O2]+; 61.0845 [C3H10N]+ ; 60.0812 [C3H10N]+ |
|  | ^13^C [M+2+H]^+^ | 220.1451 | 68.64 | C10H19NO4 | 161.0718 ; 160.0684 ; 87.032 ; 86.0318 ; 85.0285 |
|  | ^13^C [M+3+H]^+^ | 221.1486 | 68.562 | C10H19NO4 | 162.0751 ; 161.0718 ; 160.0684 ; 88.0385 ; 87.0351 ; 86.0318 ; 85.0284 ; 62.0878 ; 61.0845 ; 60.0812 |
| Purine nucleoside derivative | ^12^C [M+H]^+^ | 266.088 | 80.64 | C10H11N5O4 | 266.0881 [C10H11N5O4]+ ; 136.0619 [C5H5N5]+ ; 131.0341 [C5H6O4]+ ; 113.0233 [C5H4O3]+ ; 69.0338 [C4H4O]+ |
|  | ^13^C [M+1+H]^+^ | 267.0915 | 80.65 | C10H11N5O4 | 137.0653 ; 136.0619 ; 114.0266 ; 113.0233 |
|  | ^13^C [M+4+H]^+^ | 270.01015 | 80.66 | C10H11N5O4 | 140.0756 ; 139.0721 ; 138.0686 ; 137.0653 ; 115.0301 |
| Pyroglutamyl-leucine ([Pyro-Glu]-Leu) | ^12^C [M+H]^+^ | 243.1329 | 127.8 | C11H18N2O4 | 197.1284 [C10H17N2O2]+ ; 132.1018 [C6H14NO2]+ ; 127.0501 [C5H7N2O2]+ ; 86.0963 [C5H12N]+ ; 84.0443 [C4H6NO]+ |
|  | ^13^C [M+1+H]^+^ | 244.1363 | 121.8 | C11H18N2O4 | 198.1315 ; 133.1051 ; 87.0996 ; 86.0962 ; 85.0476 ; 84.0442 |
|  | ^13^C [M+2+H]^+^ | 245.1399 | 123 | C11H18N2O4 | 199.1352 ; 134.1086 ; 133.1053 ; 88.1030 ; 87.0997 ; 85.0448 ; 84.04438 |
| Seryl-leucine | ^12^C [M+H]^+^ | 219.1337 | 118.14 | C9H18N2O4 | 201.1237 [C9H16N2O3]+ ; 148.0603 [C5H9NO4]+ ; 173.1284 [C8H16N2O2]+ ; 132.1020 [C6H13NO2]+ ; 86.0965 [C5H11N]+ ; 60.0448 [C2H5NO]+ |
|  | ^13^C [M+3+H]^+^ | 222.1439 | 118.15 | C9H18N2O4 | 204.1334 ; 176.1385 ; 135.112 ; 134.1087 ; 89.1064 ; 88.031 ; 87.0998 |
|  | ^13^C [M+5+H]^+^ | 224.1506 | 118.15 | C9H18N2O4 | 206.1406 ; 178.1452 ; 177.1418 ; 136.1155 ; 135.1121 ; 90.1098 ; 89.1064 ; 62.0515 ; 61.0481 |
|  | ^13^C [M+6+H]^+^ | 225.1536 | 118.16 | C9H18N2O4 | 207.143 ; 179.1487 ; 178.145 ; 137.1188 ; 136.1154 ; 90.1098 ; 89.1064 ; 62.05148 ; 61.0481 |
| Tryptophylproline  (Try-Pro) | ^12^C [M+H]^+^ | 302.1496 | 181.884 | C16H19N3O3 | 285.1228 [C16H17N2O3]+ ; 170.0597 [C11H8NO]+ ; 159.0914 [C10H11N2]+ ; 144.0807 [C10H10N]+ ; 116.0705 [C5H10NO2]+ ; 114.054 [C5H8NO2]+ |
| Tyrosylproline  (Tyr-Pro) | ^12^C [M+H]^+^ | 279.1335 | 143.4 | C14H18N2O4 | 262.1075 [M-H2O+H]+ ; 136.0757 [C8H10NO]+ ; 116.0705 [C5H10NO2]+ ; 70.0654 [C4H8N]+ |
|  | ^13^C [M+3+H]^+^ | 282.1435 | 143.4 | C14H18N2O4 | 265.1174 ; 139.0858 ; 138.0825 ; 137.0791 ; 118.0773 ; 117.0739 ; 116.0705 ; 72.0721 ; 71.0687 ; 70.0654 |
|  | ^13^C [M+4+H]^+^ | 283.1465 | 141.6 | C14H18N2O4 | Isotopic pattern of M+4+H^+^ |
|  | ^13^C [M+5+H]^+^ | 284.15 | 145.08 | C14H18N2O4 | Isotopic pattern of M+5+H^+^ |
|  | ^13^C [M+7+H]^+^ | 286.1552 | 142.2 | C14H18N2O4 | Isotopic pattern of M+7+H^+^ |
| Unidentified  sesquiterpene lactone | ^12^C [M+H]^+^ | 249.148 | 286.2 | C15H20O3 | 231.1375 [M-H2O+H]+ ; 213.1272 [M-2H2O+H]+; 203.143 ; 185.1324 ; 137.0598 ; 111.044 ; 107.0854 ; 81.0699 |
|  | ^13^C [M+2+H]^+^ | 251.1555 | 285.282 | C15H20O3 | 215.1332 ; 205.149 ; 187.1387 ; 139.0661 ; 138.0628 ; 137.0595 ; 112.0471 ; 108.0886 ; 82.0731 |
|  | ^13^C [M+3+H]^+^ | 252.158 | 284.5 | C15H20O3 | Isotopic pattern of M+3+H^+^ |
|  | ^13^C [M+4+H]^+^ | 253.161 | 284.09 | C15H20O3 | Isotopic pattern of M+4+H^+^ |
|  | ^13^C [M+5+H]^+^ | 254.165 | 284.15 | C15H20O3 | Isotopic pattern of M+5+H^+^ |
|  | ^13^C [M+8+H]^+^ | 257.174 | 286.45 | C15H20O3 | Isotopic pattern of M+8+H^+^ |
|  | ^13^C [M+11+H]^+^ | 260.185 | 284.26 | C15H20O3 | Isotopic pattern of M+11+H^+^ |
| Valerylcarnitine or isovalerylcarnitine | ^12^C [M+H]^+^ | 246.1695 | 168.6 | C12H23NO4 | 187.0963 [C9H15O4]+ ; 85.0284 [C4H5O2]+ ; 60.08114 [C3H10N] |
|  | ^13^C [M+1+H]^+^ | 247.1726 | 164.4 | C12H23NO4 | 188.0995 ; 86.0316 ; 85.0283 ; 60.0811 |
|  | ^13^C [M+4+H]^+^ | 250.183 | 165.398 | C12H23NO4 | Isotopic pattern of M+4+H^+^ |
| Valylproline | ^12^C [M+H]^+^ | 215.1388 | 85.62 | C10H18N2O3 | 116.0706 [C5H9NO2]+ ; 72.0810 [C4H9N]+ ; 70.0654 [C4H7N]+ ; 55.0547 [C4H6]+ |
| Valylvaline | ^12^C [M+H]^+^ | 217.1543 | 83.76 | C10H20N2O3 | 171.1493 [C9H18N2O]+ ; 118.0865 [C5H11NO2]+ ; 72.0810 [C4H9N]+ ; 116.0705 [C5H9NO2]+ |
|  | ^13^C [M+4+H]^+^ | 221.163 | 95.2748 | C10H20N2O3 | Isotopic pattern of M+4+H^+^ |

**Table S2**. Putative annotation of the unlabeled and labeled compounds from the phytoplankton-derived DOM mix given to the natural diazotrophic communities from the ZIC-HILIC LC-ESI-(+)-MS/MS analyses. All compounds were detected with [M+H]^+^ adducts. ^a^Green and red colors indicate ^12^C and ^13^C isotopes respectively.

| Putative annotation | Isotope*^a^* | m/z | RT (sec) | Formula | MS/MS fragmentation patterns |
| --- | --- | --- | --- | --- | --- |
| Arginine | ^12^C [M+H]^+^ | 175.1191 | 347.4 | C6H14N4O2 | 158.0924 [C6H14N4O]+ ; 130.0977 [C5H14N4]+ ; 116.0707 [C5H10NO2] ; 70.0654 ; 60.0561 |
|  | ^13^C [M+1+H]^+^ | 176.1224 | 346.8 | C6H14N4O2 | 159.0958 ; 131.1011 ; 117.0741 ; 71.0688 ; 60.0561 |
| Arginyl-Valine | ^12^C [M+H]^+^ | 274.1873 | 355.8 | C11H23N5O3 | 257.1609 [C11H24N5O2]+ ; 215.1392 [C10H19N2O3]+ ; 175.1192 [C7H15N2O3]+ ; 70.0655 [C4H8N]+ |
|  | ^13^C [M+1+H]^+^ | 275.1906 | 355.2 | C11H23N5O3 | 258.1642 ; 176.1225 ; 72.0811 ; 70.0655 |
|  | ^13^C [M+2+H]^+^ | 276.194 | 355.5 | C11H23N5O3 | 259.1675 ; 177.1259 ; 176.1225 ; 73.0844 ; 72.0811 ; 71.0688 |
|  | ^13^C [M+3+H]^+^ | 277.1974 | 354.138 | C11H23N5O3 | 260.171 ; 177.1259 ; 176.122673.0844 |
| Dehydroadenosine | ^12^C [M+H]^+^ | 2660883 | 104.4 | C10H11N5O4 | 136.062 [C5H6N5]+ ; 113.0234 [C5H5O3]+ ; 69.0338 |
|  | ^13^C [M+1+H]^+^ | 267.0917 | 104.4 | C10H11N5O4 | 137.0654 ; 136.0621 ; 114.0268 ; 113.0234 ; 73.3163 ; 69.0338 |
| Deoxyguanosine | ^12^C [M+H]^+^ | 268.104 | 184.2 | C10H13N5O4 | 152.0566 [C5H5N5O]+ ; 135.0302 ; 117.0546 [C5H9O3]+ ; 99.044 [C5H7O2]+ ; 63.2644 |
|  | ^13^C [M+1+H]^+^ | 269.107 | 189 | C10H13N5O4 | 153.06 ; 152.0567 ; 138.0686 ; 137.0653 ; 136.062 ; 129.1023 |
| Trigonelline | ^12^C [M+H]^+^ | 138.0551 | 203.4 | C7H7NO2 | 138.0551 [M+H]+ ; 94.0651 [C6H8N]+ |
| Acetylcarnitine | ^12^C [M+H]^+^ | 204.1233 | 257.4 | C9H17NO4 | 145.0498 [C6H9O4]+ ; 85.0285 [C4H5O2]+; 60.0812 [C3H10N]+ |
|  | ^13^C [M+2+H]^+^ | 206.1298 | 194.4 | C9H17NO4 | 147.0564 ; 146.053 ; 87.03513 ; 86.0318 ; 85.0285 |
|  | ^13^C [M+3+H]^+^ | 207.1333 | 2586 | C9H17NO4 | 1480597 ; 147.0564 ; 146.053 ; 88.0385 ; 87.0351 ; 86.0318 ; 85.0285 |
| Propyonylcarnitine | ^12^C [M+H]^+^ | 218.1388 | 192.18 | C10H19NO4 | 159.0652 [C7H11O4]+ ; 85.02851 [C4H5O2]+ ; 60.08123 [C3H10N]+ |
| Choline | ^12^C [M+H]^+^ | 104.107 | 217.8 | C5H14NO+ | 60.0812 [C3H10N]+ |
|  | ^13^C [M+1+H]^+^ | 105.1104 | 217.2 | C5H14NO+ | 61.08453 |
| DMSP | ^12^C [M+H]^+^ | 135.0475 | 279.6 | C5H10O2S | 73.0286 [C3H5O2]+ ; 63.0267 [C2H7S]+ ; 55.0183 [C3H3O]+ |
|  | ^13^C [M+1+H]^+^ | 136.0509 | 279 | C5H10O2S | 74.0319 ; 73.0286 ; 64.03 ; 63.0267 ; 56.0216 |
|  | ^13^C [M+2+H]^+^ | 137.0544 | 279.6 | C5H10O2S | 75.0353 ; 74.032 ; 64.03 ; 63.0267 |

##

## Supplementary Figure


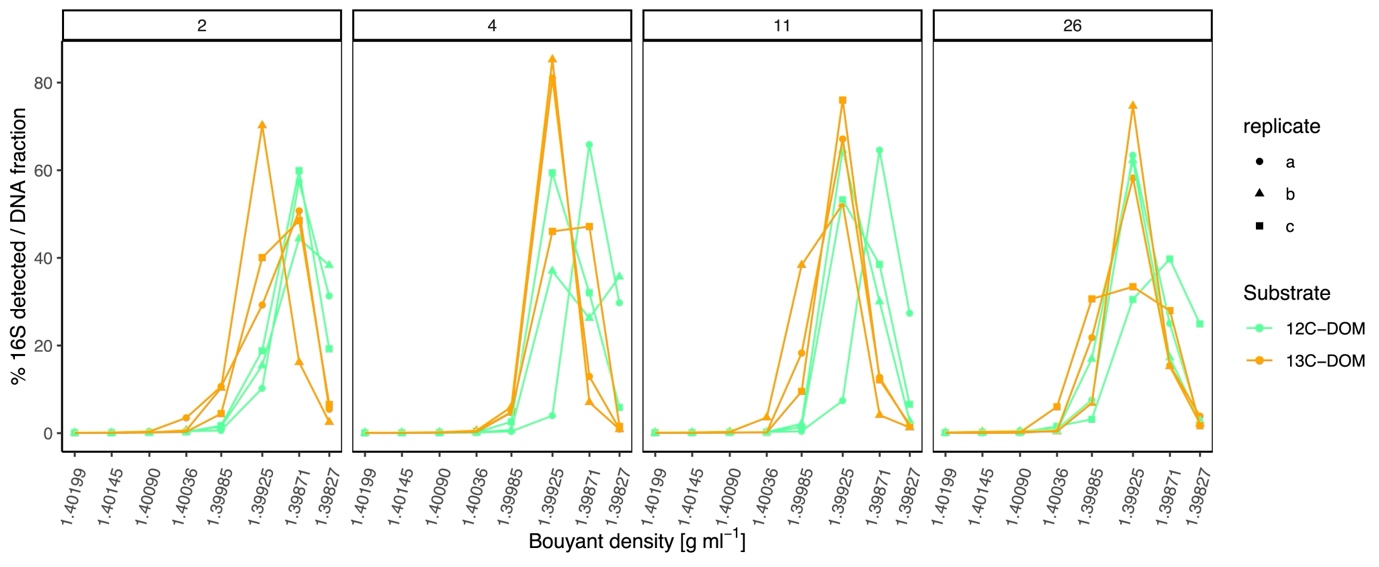


**Fig. S1**. Distribution of 16S rRNA copies along the CsCl density gradient of DNA extracted from ^13^C- (orange line) and ^12^C- (green line) DOM incubations at the four different stations. The line charts show the percentage (%) of 16S rRNA copies in each DNA fraction (different densities; g ml^-1^) compared to total 16S copies (sum of abundances at different fractions) measured by 16S qPCR assay. Based on this data the following densities 1.40036 g ml^−1^, 1.39985 g ml^−1^, 1.39925 g ml^−1^ and 1.39871 g ml^−1^, named heavy or ‘H’, medium or ‘M’, light or ‘L’ and superlight or ‘SL’, respectively were chosen for *nifH* and 16S rRNA sequencing and downstream SIP analysis (see Methods).


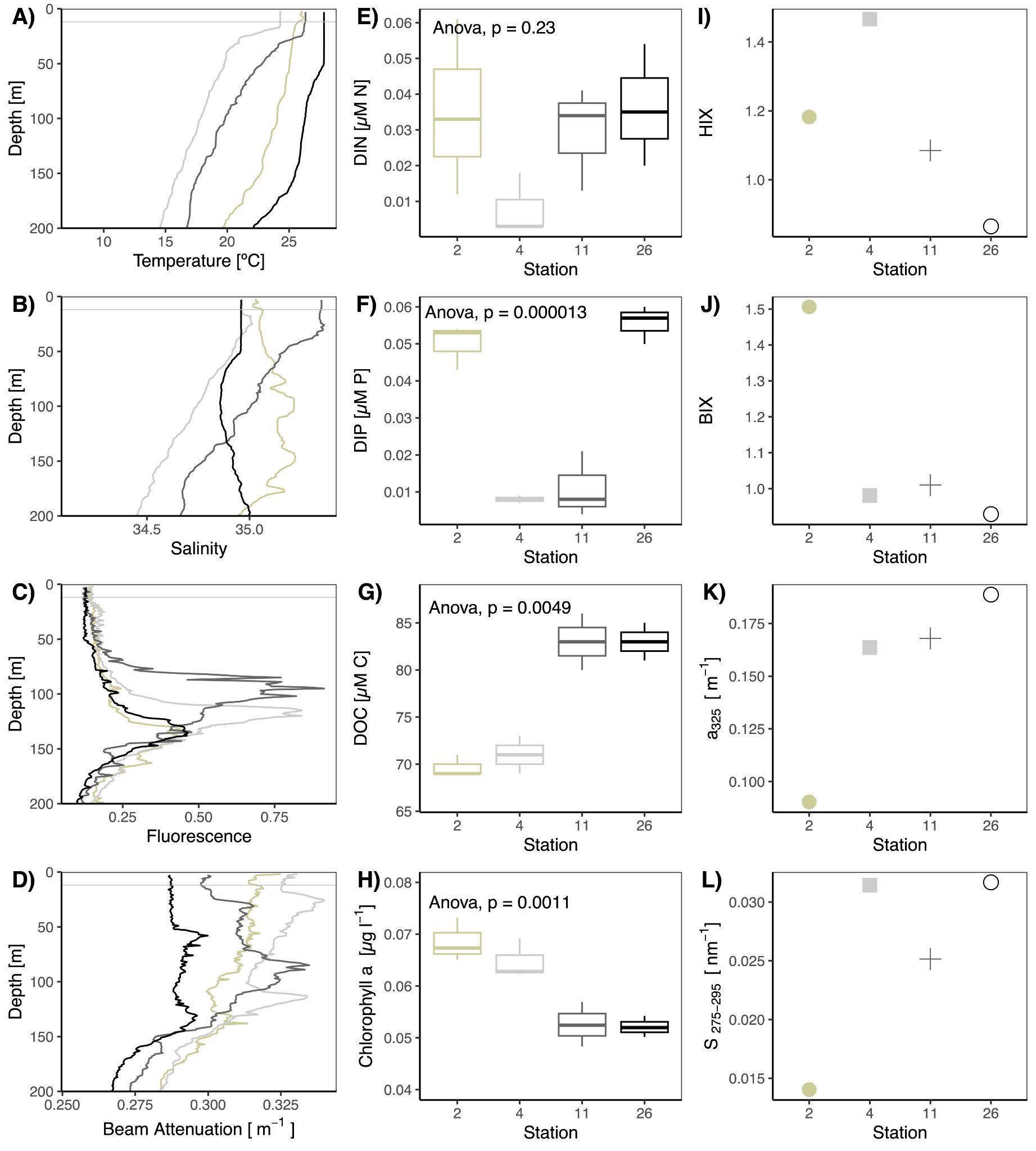


**Fig. S2.** Biogeochemical conditions. Colors and shapes represent the four different surveyed stations (2, 4, 11 and 26). The horizontal line in vertical profiles of temperature (A), salinity (B), fluorescence (C) and beam attenuation (D) measured with sensors attached to the CTD, represents the sampling depth at 15 m. Concentrations of dissolved inorganic nitrogen (DIN, E), phosphorus (DIP, F), dissolved organic carbon (DOC, G), and chlorophyll *a* (H) were analyzed from triplicate discrete initial water samples at each station. CDOM and FDOM indices including the humification index (HIX, I), biological index (BIX, J), the absorption coefficient at 325 nm (a_325_, K) and the spectral slope between 275 and 295 nm (S_275-295_ , L) were also measured from initial water samples at each station.


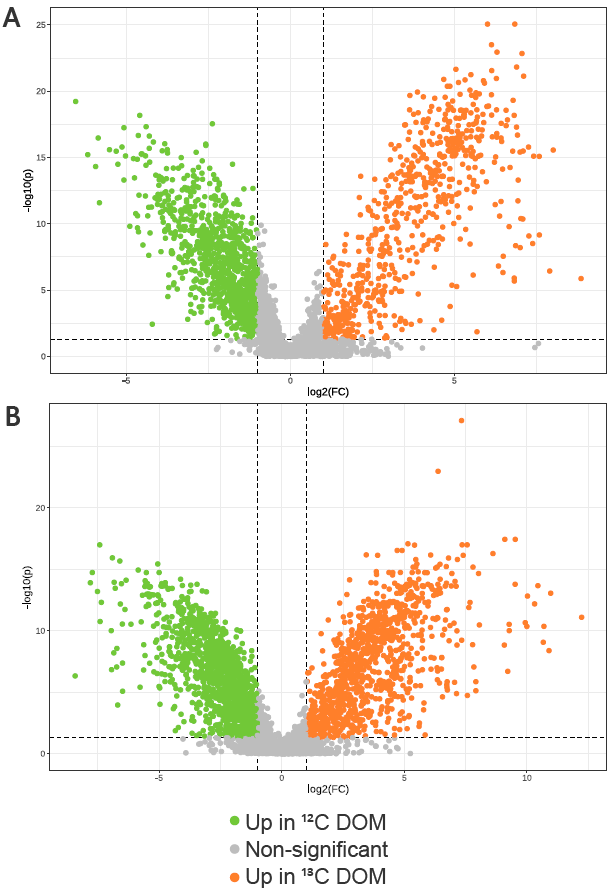


**Fig. S3.** Volcano plots showing the statistical significance and fold-change of normalized concentration of metabolites in C^12^ and C^13^ DOM pools in the C18 (A), and ZIC-HILIC (B) datasets (positive modes). Fold change (FC) threshold set at 2 and the p-value threshold set at 0.05 with false discovery rate correction.


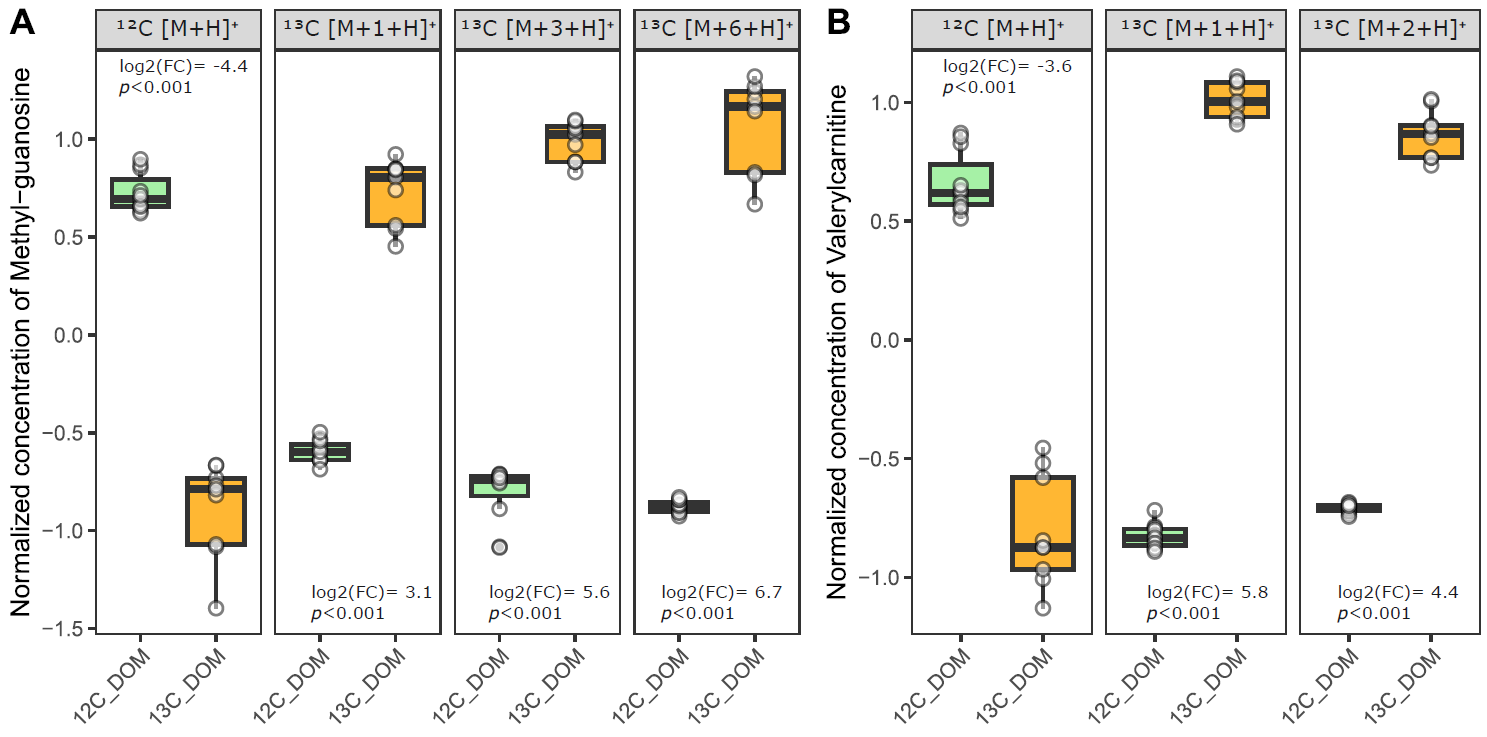


**Fig. S4.** Normalized concentration of the most discriminant isotopes between the ^12^C and ^13^C DOM pools (^12^C [M+H]^+^, and ^13^C isotopes [M+1+H]^+^, [M+2+H]^+^, [M+3+H]^+^ or [M+6+H]^+^), analyzed with the C18 positive dataset. (A) Methylguanosine, (B) Valerylcarnitine.


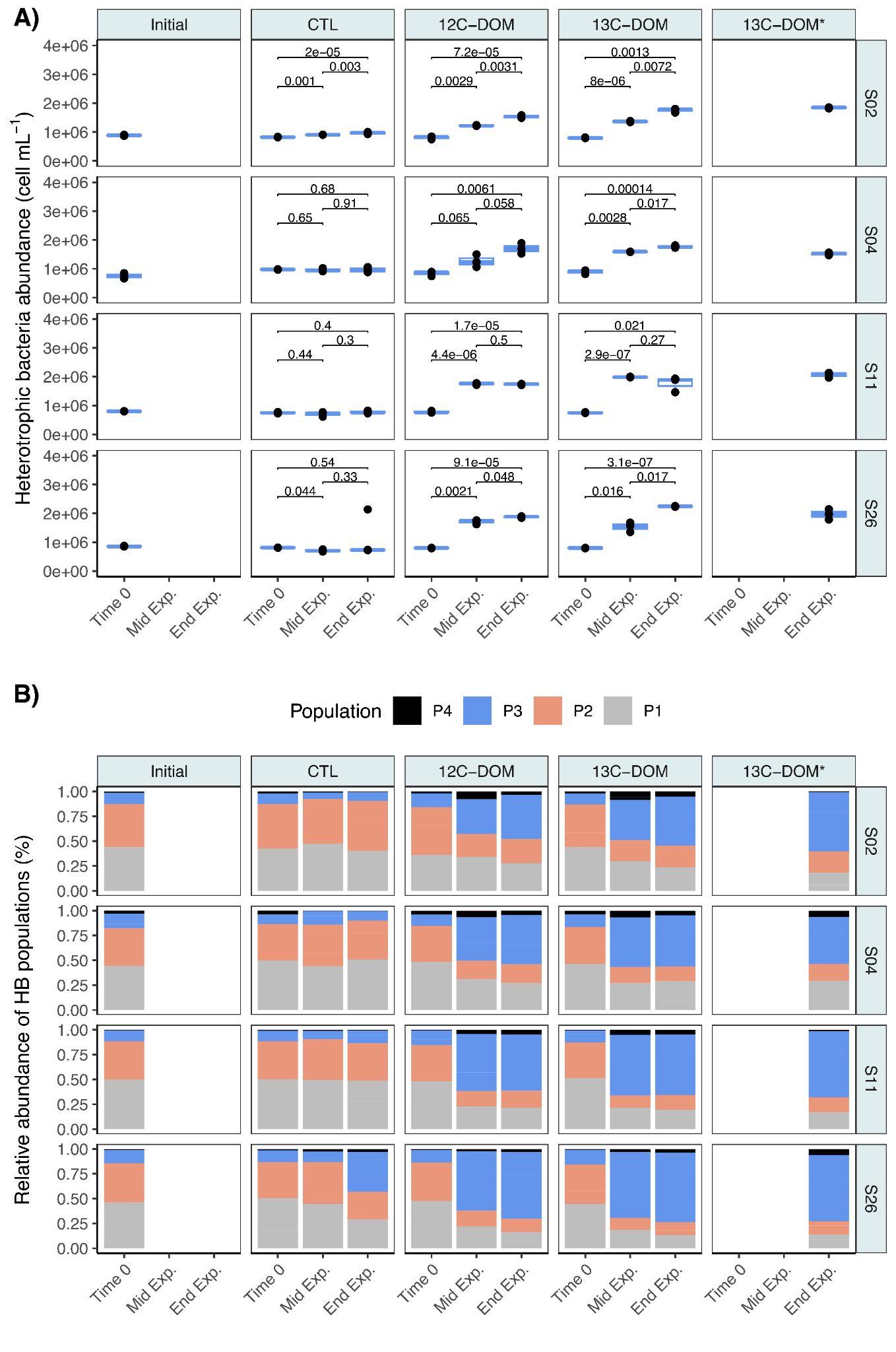


**Fig. S5.** Heterotrophic bacteria abundance (cells ml^-1^) measured by flow cytometry from each treatment bottle at different experimental points (A). Relative abundance of heterotrophic bacteria populations identified in side scatter (SSC) versus green fluorescence (FL1) cytograms(B).


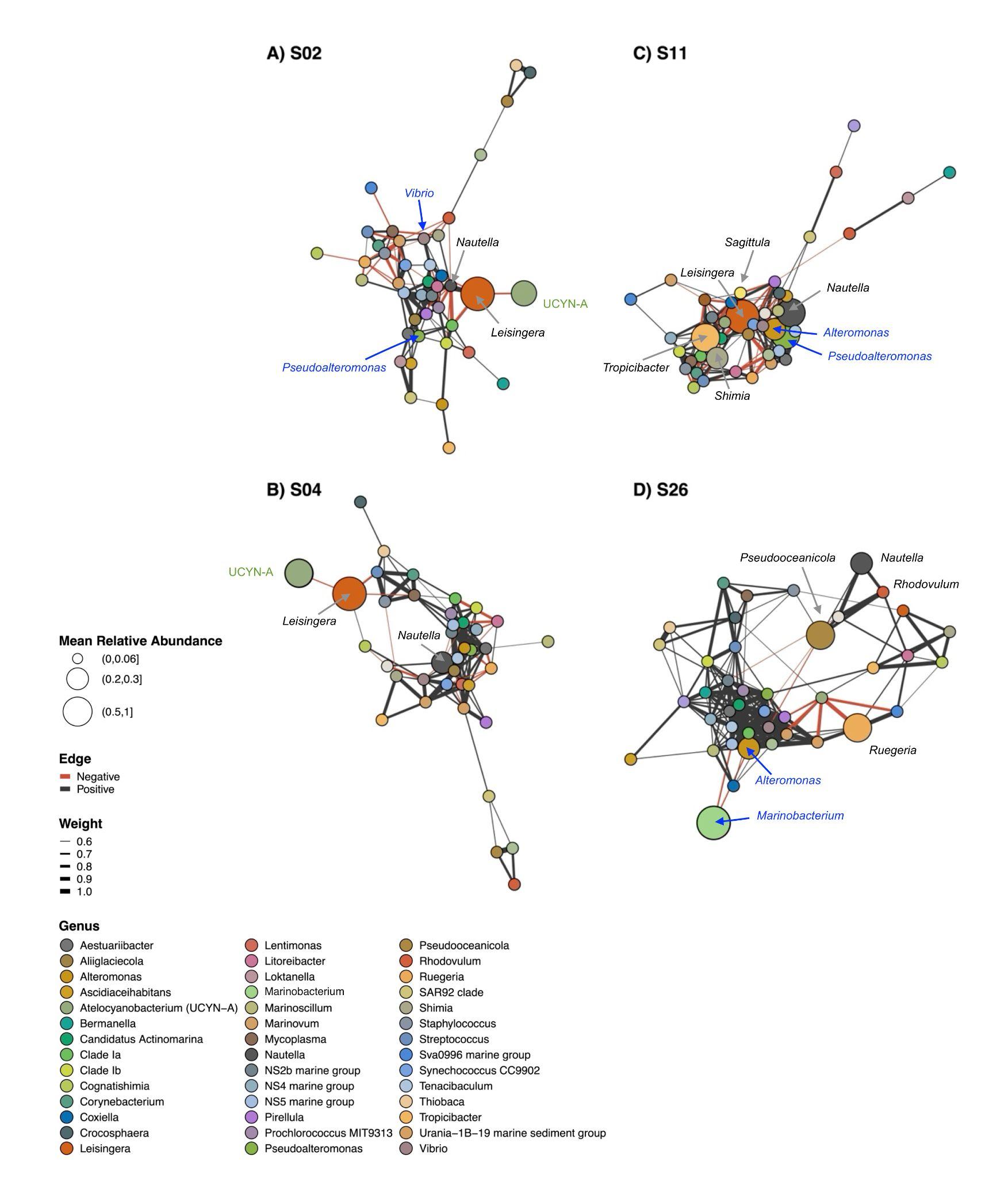


**Fig. S6. Network analysis of combined *nifH* and 16S reads for each station.** Each node represents the relative abundance of the most abundant amplicon sequence variants (ASV), grouped and colored according to the genus level**.** Spearman correlations shown are *p* < 0.05). The names of the most abundant groups of each station are shown and colored according to their higher classification into Cyanobacteria (green), Alphaproteobacteria (black) and Gammaproteobacteria (blue). Relationships between two groups are shown as lines connecting nodes (red color for positive and black color for negative relationship).

# Supplementary references

1. Aminot A, Kérouel R. Dosage automatique des nutriments dans les eaux marines: méthodes en flux continu. Editions Quae; 2007.

2. Holm-Hansen O, Lorenzen CJ, Holmes RW, Strickland JDH. Fluorometric determination of chlorophyll. ICES Journal of Marine Science. 1965;30(1):3–15.

3. Sohrin R, Sempéré R. Seasonal variation in total organic carbon in the northeast Atlantic in 2000–2001. J Geophys Res Oceans. 2005;110(C10).

4. Ferretto N, Tedetti M, Guigue C, Mounier S, Raimbault P, Goutx M. Spatio-temporal variability of fluorescent dissolved organic matter in the Rhône River delta and the Fos-Marseille marine area (NW Mediterranean Sea, France). Environmental Science and Pollution Research. 2017;24:4973–89.

5. Helms JR, Stubbins A, Ritchie JD, Minor EC, Kieber DJ, Mopper K. Absorption spectral slopes and slope ratios as indicators of molecular weight, source, and photobleaching of chromophoric dissolved organic matter. Limnol Oceanogr. 2008;53(3):955–69.

6. Helms JR, Stubbins A, Perdue EM, Green NW, Chen H, Mopper K. Photochemical bleaching of oceanic dissolved organic matter and its effect on absorption spectral slope and fluorescence. Mar Chem. 2013;155:81–91.

7. Benavides M, Martias C, Elifantz H, Berman-Frank I, Dupouy C, Bonnet S. Dissolved organic matter influences N2 fixation in the New Caledonian lagoon (Western Tropical South Pacific). Front Mar Sci. 2018;5:89.

8. Tedetti M, Longhitano R, Garcia N, Guigue C, Ferretto N, Goutx M. Fluorescence properties of dissolved organic matter in coastal Mediterranean waters influenced by a municipal sewage effluent (Bay of Marseilles, France). Environmental Chemistry. 2012;9(5):438–49.

9. Ohno T. Fluorescence inner-filtering correction for determining the humification index of dissolved organic matter. Environ Sci Technol. 2002;36(4):742–6.

10. Coble PG. Marine optical biogeochemistry: the chemistry of ocean color. Chem Rev. 2007;107(2):402–18.

11. Murphy KR, Stedmon CA, Graeber D, Bro R. Fluorescence spectroscopy and multi-way techniques. PARAFAC. Analytical methods. 2013;5(23):6557–66.

12. Zsolnay A, Baigar E, Jimenez M, Steinweg B, Saccomandi F. Differentiating with fluorescence spectroscopy the sources of dissolved organic matter in soils subjected to drying. Chemosphere. 1999;38(1):45–50.

13. Huguet A, Vacher L, Relexans S, Saubusse S, Froidefond JM, Parlanti E. Properties of fluorescent dissolved organic matter in the Gironde Estuary. Org Geochem. 2009;40(6):706–19.

14. Rippka R, Deruelles J, Waterbury JB, Herdman M, Stanier RY. Generic assignments, strain histories and properties of pure cultures of cyanobacteria. Microbiology (N Y). 1979;111(1):1–61.

15. Wang M, Carver JJ, Phelan V V, Sanchez LM, Garg N, Peng Y, et al. Sharing and community curation of mass spectrometry data with Global Natural Products Social Molecular Networking. Nat Biotechnol. 2016;34(8):828–37.

16. Moisander PH, Beinart RA, Voss M, Zehr JP. Diversity and abundance of diazotrophic microorganisms in the South China Sea during intermonsoon. ISME J. 2008;2(9):954–67.

17. Neufeld JD, Vohra J, Dumont MG, Lueders T, Manefield M, Friedrich MW, et al. DNA stable-isotope probing. Nat Protoc. 2007;2(4):860–6.

18. Zehr JP, McREYNOLDS LA. Use of degenerate oligonucleotides for amplification of the nifH gene from the marine cyanobacterium Trichodesmium thiebautii. Appl Environ Microbiol. 1989;55(10):2522–6.

19. Zehr JP, Turner PJ. Nitrogen fixation: nitrogenase genes and gene expression. Methods in microbiology. 2001;30:271–86.

20. Benavides M, Caffin M, Duhamel S, Foster RA, Grosso O, Guieu C, et al. Anomalously high abundance of Crocosphaera in the South Pacific Gyre. FEMS Microbiol Lett. 2022;369(1):fnac039.

21. Callahan BJ, McMurdie PJ, Rosen MJ, Han AW, Johnson A. JA, Holmes SP. 2016. DADA2: high-resolution sample inference from Illumina amplicon data Nature Methods. 2016;13(7):581–3.

22. Angel R, Nepel M, Panhölzl C, Schmidt H, Herbold CW, Eichorst SA, et al. Evaluation of primers targeting the diazotroph functional gene and development of NifMAP–a bioinformatics pipeline for analyzing nifH amplicon data. Front Microbiol. 2018;9:703.

23. Wang Q, Quensen III JF, Fish JA, Kwon Lee T, Sun Y, Tiedje JM, et al. Ecological patterns of nifH genes in four terrestrial climatic zones explored with targeted metagenomics using FrameBot, a new informatics tool. mBio. 2013;4(5):e00592-13.

24. Parada AE, Needham DM, Fuhrman JA. Every base matters: assessing small subunit rRNA primers for marine microbiomes with mock communities, time series and global field samples. Environ Microbiol. 2016;18(5):1403–14.

25. Apprill A, McNally S, Parsons R, Weber L. Minor revision to V4 region SSU rRNA 806R gene primer greatly increases detection of SAR11 bacterioplankton. Aquatic Microbial Ecology. 2015;75(2):129–37.

26. Montoya JP, Voss M, Kahler P, Capone DG. A simple, high-precision, high-sensitivity tracer assay for N (inf2) fixation. Appl Environ Microbiol. 1996;62(3):986–93.

27. Kana TM, Darkangelo C, Hunt MD, Oldham JB, Bennett GE, Cornwell JC. Membrane inlet mass spectrometer for rapid high-precision determination of N2, O2, and Ar in environmental water samples. Anal Chem. 1994;66(23):4166–70.

28. Gradoville MR, Bombar D, Crump BC, Letelier RM, Zehr JP, White AE. Diversity and activity of nitrogen‐fixing communities across ocean basins. Limnol Oceanogr. 2017;62(5):1895–909.

29. Martínez-Pérez C, Mohr W, Schwedt A, Dürschlag J, Callbeck CM, Schunck H, et al. Metabolic versatility of a novel N2-fixing Alphaproteobacterium isolated from a marine oxygen minimum zone. Environ Microbiol. 2018;20(2):755–68.

30. Howat AM, Vollmers J, Taubert M, Grob C, Dixon JL, Todd JD, et al. Comparative genomics and mutational analysis reveals a novel XoxF-utilizing methylotroph in the Roseobacter group isolated from the marine environment. Front Microbiol. 2018;9:766.

31. Delmont TO, Pierella Karlusich JJ, Veseli I, Fuessel J, Eren AM, Foster RA, et al. Heterotrophic bacterial diazotrophs are more abundant than their cyanobacterial counterparts in metagenomes covering most of the sunlit ocean. ISME J. 2022;16(4):927–36.

32. Han SB, Wang RJ, Yu XY, Su Y, Sun C, Fu GY, et al. Marinobacterium zhoushanense sp. nov., isolated from surface seawater. Int J Syst Evol Microbiol. 2016;66(9):3437–42.

33. Kim H, Choo YJ, Song J, Lee JS, Lee KC, Cho JC. Marinobacterium litorale sp. nov. in the order Oceanospirillales. Int J Syst Evol Microbiol. 2007;57(7):1659–62.

34. Durán-Viseras A, Castro DJ, Reina JC, Béjar V, Martínez-Checa F. Taxogenomic and metabolic insights into Marinobacterium ramblicola sp. nov., a new slightly halophilic bacterium isolated from Rambla Salada, Murcia. Microorganisms. 2021;9(8):1654.
